# Analysis of the laccase gene family and miR397-/miR408-mediated posttranscriptional regulation in *Salvia miltiorrhiza*

**DOI:** 10.7717/peerj.7605

**Published:** 2019-08-29

**Authors:** Caili Li, Dongqiao Li, Hong Zhou, Jiang Li, Shanfa Lu

**Affiliations:** Institute of Medicinal Plant Development, Chinese Academy of Medical Sciences & Peking Union Medical College, Beijing, China

**Keywords:** *Salvia miltiorrhiza*, Laccase, Phenolic compound, Gene expression, miR397, miR408, Laccase gene family, Phenolic compounds

## Abstract

*Salvia miltiorrhiza* is one of the most commonly used traditional Chinese medicine materials. It contains important bioactive phenolic compounds, such as salvianolic acids, flavonoids and anthocyanins. Elucidation of phenolic compound biosynthesis and its regulatory mechanism is of great significance for *S. miltiorrhiza* quality improvement. Laccases (LACs) are multicopper-containing enzymes potentially involved in the polymerization of phenolic compounds. So far, little has been known about *LAC* genes in *S. miltiorrhiza*. Through systematic investigation of the whole genome sequence and transcriptomes of *S. miltiorrhiza*, we identified 65 full-length *SmLAC* genes (*SmLAC1*–*SmLAC65*). Phylogenetic analysis showed that 62 of the identified SmLACs clustered with LACs from *Arabidopsis* and *Populus trichocarpa* in seven clades (C1–C7), whereas the other three fell into one *S. miltiorrhiza*-specific clade (C8). All of the deduced SmLAC proteins contain four conserved signature sequences and three typical Cu-oxidase domains, and gene structures of most *LACs* from *S. miltiorrhiza*, *Arabidopsis* and *P. trichocarpa* were highly conserved, however *SmLACs* encoding C8 proteins showed distinct intron-exon structures. It suggests the conservation and diversity of plant *LACs* in gene structures. The majority of *SmLACs* exhibited tissue-specific expression patterns, indicates manifold functions of *SmLACs* played in *S. miltiorrhiza*. Analysis of high-throughput small RNA sequences and degradome data and experimental validation using the 5′ RACE method showed that 23 *SmLACs* were targets of Smi-miR397. Among them, three were also targeted by Smi-miR408. It suggests the significance of miR397 and miR408 in posttranscriptional regulation of *SmLAC* genes. Our results provide a foundation for further demonstrating the functions of *SmLACs* in the production of bioactive phenolic compounds in *S. miltiorrhiza*.

## Introduction

Laccases (LACs, benzenediol: oxygen oxidoreductases, EC 1.10.3.2) are blue multicopper oxidases widely existing in plants, fungi, bacteria and insects. Laccases proteins have three catalytic sites, named T1, T2 and T3, respectively. When a substrate is oxidized at T1, an electron is released and transferred to T2/T3 trinuclear copper cluster (TNC) via cys-his pathway, consequently the free hydrogens are combined with molecule oxygens (O_2_) and reduced to water molecules (H_2_O). The resulting oxidized products, which are usually free-radical species, may be involved in subsequent radical–radical coupling (Mayer & Staples, 2002; Giardina et al., 2010) or cross-linking with other substances, including extracellular molecules (Mattinen et al., 2005; Giardina et al., 2010).

So far, genes encoding LACs have been identified from various plant species, such as *Arabidopsis* (McCaig, Meagher & Dean, 2005; Turlapati et al., 2011), rice (Liu et al., 2017), tobacco (Richardson & McDougall, 1997), ryegrass (Gavnholt, Larsen & Rasmussen, 2002), cotton (Balasubramanian et al., 2016), poplar (LaFayette, Eriksson & Dean, 1999; Lu et al., 2013), sycamore maple (Sterjiades, Dean & Eriksson, 1992) and pear (Cheng et al., 2019). *LAC* genes present as a multi-gene family in plants. For instance, *Arabidopsis* has 17 annotated *LACs* (McCaig, Meagher & Dean, 2005; Turlapati et al., 2011), rice has 30 (Liu et al., 2017) and *P. trichocarpa* has 49 (Lu et al., 2013). Phylogenetic analysis of LACs from *Arabidopsis*, rice and/or *P. trichocarpa* showed that plant LACs could be divided into six clades (C1–C6) (McCaig, Meagher & Dean, 2005; Turlapati et al., 2011). *Arabidopsis* AtLACs and *P. trichocarpa* PtLACs distributed in all of the six clades, whereas rice OsLACs distributed in five clades (McCaig, Meagher & Dean, 2005; Turlapati et al., 2011; Liu et al., 2017; Lu et al., 2013).

It has been shown that plant LACs are involved in lignin (Berthet et al., 2011; Brown et al., 2005) and flavonoid biosynthesis (Pourcel et al., 2005). Laccases could polymerize lignin monomers in vitro (Freudenberg, 1959). The expression of some *LAC* genes was correlated with lignin deposition in xylem (Sterjiades, Dean & Eriksson, 1992; Dean & Eriksson, 1994; Bao et al., 1993; Schuetz et al., 2014). In *Arabidopsis*, eight out of 17 *AtLACs* were highly expressed in the inflorescence stems, where lignin was deposited. Double mutation of two stem-expressed *AtLACs*, *lac4* and *lac17*, resulted in significant decrease of lignin content (Berthet et al., 2011), and in a *lac11lac4lac17* triple-mutant, root lignin content was extremely low (Zhao et al., 2013). This suggests that some plant LACs are involved in lignin polymerization. In addition to lignin, LACs also participate in flavonoid biosynthesis. For instance, AtLAC15, which is also known as TRANSPARENT TESTA10 (TT10), may function as an oxidase in the oxidative polymerization of flavonoids. Mutation of *AtLAC15* resulted in a delay in developmentally determined browning of the seed coat and an accumulation of more epicatechin monomers and soluble proanthocyanidins than wild-type seeds (Pourcel et al., 2005).

MicroRNAs (miRNAs) are a large family of 21–24 nt endogenous small non-coding ribonucleic acid (RNAs) playing important regulatory roles in many bioprocesses (Brodersen & Voinnet, 2009; Filipowicz, Bhattacharyya & Sonenberg, 2008; Vaucheret, 2006). Various studies have shown that miRNAs, including miR397, miR408, miR528 and miR857, tightly regulate the expression of plant *LAC* genes at the posttranscriptional level. In woody *Citrus*, miR397a plays a pivotal role in the tolerance to B-toxicity by targeting *LAC17* and *LAC4* that are responsible for secondary cell-wall synthesis (Huang et al., 2016). Analysis of *Arabidopsis* transgenics plants overexpressing Ath-miR397 showed that Ath-miR397b could regulate lignin content and seed number through modulation of *AtLAC4* (Wang et al., 2014). Overexpression of Osa-miR397 in rice significantly downregulated its target, *OsLAC*, and improved rice yield by increasing grain size and promoting panicle branching (Zhang et al., 2013). In the woody plant *P. trichocarpa*, Ptr-miR397a acted as a negative regulator of a subset of *LAC* genes involved in lignin polymerization during wood formation (Lu et al., 2013). Overexpression of Ptr-miR397a in *P. trichocarpa* severely reduced *LAC* gene expression, total LAC activity and lignin content (Lu et al., 2013). Overexpression of *PbrmiR397a* and simultaneous silencing of three *LAC* genes reduced the lignin content and stone cell number in pear fruit (Xue et al., 2019).

*Salvia miltiorrhiza* Bunge, known as danshen in Chinese, is one of the most important materials of traditional Chinese medicines (TCMs). It is also an emerging model system for medicinal plant biology (Song et al., 2013). The whole genome of *S. miltiorrhiza*has recently been sequenced (Zhang et al., 2015; Xu et al., 2016). Phenolic compounds, such as salvianolic acids, flavonoids and anthocyanins, are important bioactive substances in *S. miltiorrhiza* (Deng et al., 2018; Deng & Lu, 2017; Hou et al., 2013). Elucidating the biosynthetic mechanism of these substances is of great significance for *S. miltiorrhiza* quality improvement. However, no efforts have been taken to identify and characterize the *LAC* genes in *S. miltiorrhiza*, although the importance of *LACs* has been shown in a variety of plants. In this study, using bioinformatics approaches, we identified 65 *SmLAC* genes from the *S. miltiorrhiza* genome. Gene structures, sequence characteristics and gene expression patterns were analyzed. Computational and experimental analysis showed that Smi-miR397 and Smi-miR408 were involved in posttranscriptional regulation of *SmLACs*. The results provide useful information for further elucidating *SmLAC* functions.

## Materials and Methods

### Plant materials

Two-year-old plants of *S. miltiorrhiza* Bunge (line 99-3) were used as a source of plant tissues. The plants were grown in a field nursery under natural conditions at the Institute of Medicinal Plant Development, Beijing, China. Flowers, leaves, stems and roots were harvested in June and then immediately frozen in liquid nitrogen for nucleic acid isolation.

### Gene prediction

Protein sequences of 17 *A. thaliana* LACs described by McCaig, Meagher & Dean (2005) were downloaded from the *Arabidopsis* Information Resource (TAIR, http://www.Arabidopsis.org/). Amino acid sequences of 49 *P. trichocarpa* LACs (PtLACs) were downloaded from the *P. trichocarpa* genome assembly v3.0 (https://phytozome.jgi.doe.gov/pz/portal.html#!info?alias=Org_Ptrichocarpa_er) (Lu et al., 2013). BLAST analysis of AtLACs and PtLACs against the genome database for *S. miltiorrhiza* line 99-3 (Xu et al., 2016) was carried out using BLASTp with value ≤10^−10^. Gene models were predicted from retrieved genomic DNA sequences through alignment with *LAC* genes from other plants and RNA-Seq data of *S. miltiorrhiza* transcriptome (http://www.ncbi.nlm.nih.gov) using the BLASTx and BLASTn program, respectively. The predicted gene models were checked for accuracy by comparison with another genome database for *S. miltiorrhiza* (http://www.ndctcm.org/shujukujieshao/2015-04-23/27.html) (Zhang et al., 2015). All of the identified laccase sequences were analyzed for conserved domain by blast against the Pfam database (http://pfam.janelia.org). The obtained open reading frame (ORF) sequences for *SmLAC1*–*SmLAC65* are available in Data S1.

### RNA extraction, reverse transcription and cDNA cloning

Total RNA was extracted from *S. miltiorrhiza* tissues using the plant total RNA isolation kit (Aidlab, Beijing, China). Ribonucleic acid quality was examined by gel electrophoresis and NanoDrop 2000C spectro-photometer (Thermo Scientific, Waltham, MA, USA). For 5′ and 3′ RACE, 10µg total RNA was converted into cDNA using SMARTer® RACE 5′/3′Kit (TaKaRa Bio, Otsu, Japan). For polymerase chain reaction (PCR) amplification of coding region, 5 µg total RNA was reverse transcribed into cDNA using SuperScript Ш Reverse Transcriptase (Invitrogen, Carlsbad, CA, USA) in 20 µl volume.

Rapid amplification of 5′ and 3′cDNAs ends was carried out using the SMART^TM^ RACE cDNA amplification kit (TaKaRa Bio, Otsu, Japan). The primers used for PCR amplification are listed in Tables S1 and S2. Nesting PCR amplification was performed under the following program of touchdown PCR: 95 °C for 5 min, 5 cycles of amplification at 95 °C for 30 s and 72 °C for 3 min, 5 cycles of amplification at 95 °C for 30 s, 70 °C for 30 s and 72 °C for 1 min, 27 cycles of amplification at 95 °C for 30 s, 68 °C for 30 s and 72 °C for 1 min, followed by a final extension at 72 °C for 10 min. Nested PCR amplification was carried out under the following conditions: 95 °C for 5 min, 25 cycles of amplification at 95 °C for 30 s, TM (Based on specific primers) for 45 s and 72 °C for 1 min, followed by a final extension at 72 °C for 10 min. Polymerase chain reaction products were gel-purified and cloned into pTOPO-T vector (Aidlab Biotechnologies, Beijing, China), for sequencing by Sanger method (GENEWIZ, Beijing, China).

Full-length coding sequences were amplified by PCR using gene-specific forward and reverse primer pairs (Table S3) under the following reaction conditions: initial denaturation at 95 °C for 5 min, 25 cycles of amplification at 95 °C for 30 s, TM (Based on specific primers) for 45 s and 72 °C for 1.5 min, followed by a final extension at 72 °C for 10 min. Polymerase chain reaction products were purified using the DNA Gel extraction kit (Aidlab, Beijing, China). The purified fragments were cloned into pMD19-T vector (TaKaRa, Otsu, Japan), and then sequenced by Sanger method (GENEWIZ, Beijing, China).

### Bioinformatic analysis and phylogenetic tree construction

The theoretical isoelectric point (p*I*) and molecular weight (Mw) of SmLAC proteins were analyzed using the Compute p*I*/Mw tool on the ExPASy server (http://web.expasy.org/compute_pi/) (Bjellqvist et al., 1994). The conserved domains were identified for SmLACs using the MEME server v4.11.4 (http://meme-suite.org/tools/meme) (Bailey et al., 2009) with the minimum and maximum weight of 3 and 100 amino acid residues, respectively. Protein sequences with three Cu-oxidase domains in the LAC domain were recognized as members of the LAC family. Sequence logos were created on the WebLogo server (http://weblogo.berkeley.edu/logo.cgi) (Crooks et al., 2004). The putative signal sequences and potential glycosylation sites of the proteins were predicted using TargetP and NetNGlyc server v1.1 (http://www.cbs.dtu.dk/) (Emanuelsson et al., 2000), respectively. The default cut-off values were adopted with plant as the organism. Phylogenetic tree was constructed by the neighbor-joining (NJ) method with 1,000 bootstrap replicates using MEGA 7.0 (Kumar, Stecher & Tamura, 2016).

Intron/exon structure was determined through the comparison of the coding sequence of each *SmLAC* gene with its genomic sequence using the Gene Structure Display Server 2.0 (http://gsds.cbi.pku.edu.cn/index.php) (Hu et al., 2015). We found the 1,500 bp sequence upstream from each *SmLAC* initiation codon and used the online tool PlantCARE (http://bioinformatics.psb.ugent.be/webtools/plantcare/html/) for the prediction of *cis*-elements. SmLAC proteins were further subject to GO annotations analysis according to the Gene Ontology (GO, geneontology.org).

### Quantitative real-time reverse transcription-PCR

Quantitative real-time reverse transcription-PCR (qRT-PCR) analysis of *SmLACs* in flowers, leaves, stems and roots of 2-year-old *S. miltiorrhiza* plants were performed using gene-specific primers listed in Table S4. Polymerase chain reaction was performed in a 20 µl volume with 100 ng diluted cDNA, 10 µM forward primer, 10 µM reverse primer and 10 µl 2 × SYBR Premix Ex Taq II (TaKaRa Bio, Otsu, Japan). Quantitative real-time reverse transcription-PCR was carried out with a CFX96 real-time PCR machine (BIO-RAD, American). *SmUBQ10* was used as a reference gene (Li et al., 2015). The expression of Smi-miR397.1, Smi-miR397.2 and Smi-miR408 was analyzed using Mir-X miRNA qRT-PCR SYBR Kit (TaKaRa, Otsu, Japan). The primers were listed in Table S4. Polymerase chain reaction cycling began with template denaturation and hot start Taq activation at 95 °C for 30 s, then 40 cycles of 95 °C for 15 s, and 60 °C for 30 s and 72 °C for 30 s extension step. All of the qRT-PCRs were repeated for three biological and three technical replicates. The 2^−ΔΔCt^ method was used for evaluating gene expression levels. Three biological replicates were standardized as described previously (Willems, Leyns & Vandesompele, 2008). All data were subjected to variance (ANOVA) analysis and the results were presented as the mean ± SD. *P* < 0.05 was considered as statistically significant.

### Identification of SmLACs with perfect or near-perfect complementary sequences to miRNAs

Small RNAs sequences from roots (SRX686651), stems (SRX686652), leaves (SRX686653) and flowers (SRX686654) of *S*. *miltiorrhiza*were downloaded from SRA database (Xu et al., 2014). The complementarity of *SmLACs* to small RNAs was searched using psRNATarget (http://plantgrn.noble.org/psRNATarget/) (Dai & Zhao, 2011) with default parameters. The maximum expectation value was 3.0, length for complementarity scoring was 19 bp and the target accessibility-allowed maximum energy to unpair the target site of 25.0 was applied. Flanking length around target site for target accessibility analysis (UPE calculation) was 17 bp upstream and 13 bp downstream and range of central mismatch causing translational inhibition was set 10–11 nucleotides. The predicted small RNAs were aligned with *S*. *miltiorrhiza* 99-3 genome using Bowtie (Langmead et al., 2009). Secondary structures were predicted on the mfold web server (Zuker, 2003).

### Degradome and experimental verification of miRNA-directed cleavage of SmLACs

Degradome data were aligned to *SmLACs* using SOAP2.0 software (Li et al., 2009) (http://soap.genomics.org.cn/) to define the coverage rate. Alignments with scores not exceeding four and no mismatches at the cleavage site (between the 10th and 11th nucleotides) were considered to be products of miRNA cleavage. Experimental validation of miRNA targets was carried out using the modified RNA ligase-mediated rapid amplification of 5′cDNAs method (5′RLM RACE) as described (Li et al., 2017). Gene-specific primers (GSP and NGSP) were designed by Premier 5.0 software (Tables S5 and S6). Two rounds of PCR were performed. Initial PCR reactions were done with the 5′RACE outer primer and the complementary GSP. The nested PCR was performed with the 5′RACE inner primer and the NGSP. Nested PCR products were analyzed on a 1.5% agarose gel. Positive PCR products were cloned into pMD-19T vector (TaKaRa, Otsu, Japan) and sequenced.

## Results

### Identification of 65 SmLAC genes in *S. miltiorrhiza*

Blast analysis of 17 AtLACs from *Arabidopsis* and 49 PtLACs from *P. trichocarpa* against the current assembly of the whole *S. miltiorrhiza* genome sequence (Xu et al., 2016) identified 85 genomic loci putatively encoding LACs. Genomic DNA sequences surrounding the 85 loci were retrieved and computationally predicted for gene models by alignment with RNA-seq data of *S*. *miltiorrhiza* (http://www.ncbi.nlm.nih.gov/sra) and LACs identified in other species (www.ncbi.nlm.nih.gov/blast/). Errors from genome sequencing and assembly were corrected after sequence alignment. Since plant LACs consist of three conserved Cu-oxidase domains with signature sequences (Diamantidis et al., 2000), the gene models predicted were analyzed for conserved domains by blast analysis against the Pfam database. The results showed that 49 gene models encoded full-length LACs, 16 were laccase gene fragments, whereas the other 20 coded for L-ascorbate oxidase proteins, which were excluded from further analysis. We then obtained full-length coding sequences (CDSs) of the 16 partial *SmLACs* using experimental approaches, including 5′ RACE, 3′ RACE and PCR amplification of coding regions. Finally, a total of 65 *SmLACs* were identified (Data S1). They were named *SmLAC1*–*SmLAC65*, respectively (Table 1). Sequence feature analysis showed that the theoretical p*I* of deduced *S. miltiorrhiza* LAC proteins widely ranged from 5.23 (SmLAC21) to 9.73 (SmLAC43). The molecular weights of the SmLAC proteins range from 54.8 kDa (SmLAC7) to 70.7 kDa (SmLAC63) and the length varied between 504 (SmLAC7) to 630 (SmLAC63) amino acids.

**Table 1:** Protein sequences of SmLACs.

Name	AA Len	p*I*	Mw (Da)	Predicted subcellular location	N-Glyc	O-Glyc
SmLAC1	566	8.54	62214.94	Secretory	11	13
SmLAC2	564	8.27	62644.60	Secretory	5	10
SmLAC3	578	7.64	63022.57	Secretory	10	13
SmLAC4	559	8.87	61097.94	Secretory	11	14
SmLAC5	553	7.08	62556.68	Mitochondrion	5	10
SmLAC6	573	9.21	63120.82	Secretory	12	8
SmLAC7	504	5.96	54842.84	Secretory	9	21
SmLAC8	568	8.84	62949.54	Secretory	7	6
SmLAC9	612	5.93	68171.10	Secretory	17	12
SmLAC10	570	5.99	62138.75	Secretory	12	12
SmLAC11	542	9.05	59612.02	Secretory	11	7
SmLAC12	565	6.62	63234.93	Secretory	11	12
SmLAC13	575	8.02	65087.05	Secretory	2	10
SmLAC14	548	8.62	62462.27	Extracellular	5	6
SmLAC15	568	9.16	62486.70	Secretory	17	9
SmLAC16	577	9.17	63496.65	Secretory	15	11
SmLAC17	565	8.40	62481.15	Secretory	9	8
SmLAC18	555	9.30	61366.90	Secretory	11	13
SmLAC19	529	8.72	59939.00	Secretory	7	8
SmLAC20	573	6.79	64637.23	Secretory	4	8
SmLAC21	540	5.23	60626.67	Secretory	5	7
SmLAC22	566	8.02	64165.84	Secretory	4	9
SmLAC23	513	8.57	56859.20	Secretory	9	12
SmLAC24	550	9.09	60671.31	Secretory	8	13
SmLAC25	561	6.11	63020.84	Secretory	10	7
SmLAC26	568	8.71	62686.13	Secretory	8	15
SmLAC27	506	8.89	56727.77	Secretory	8	13
SmLAC28	562	9.41	62070.94	Secretory	11	10
SmLAC29	553	7.67	62113.75	Secretory	14	8
SmLAC30	532	6.74	60027.81	Secretory	5	12
SmLAC31	556	8.44	62176.34	Secretory	10	8
SmLAC32	570	9.19	63318.38	Secretory	8	16
SmLAC33	565	7.29	63504.00	Secretory	11	8
SmLAC34	566	9.29	60713.98	Secretory	8	26
SmLAC35	550	6.93	62153.83	Secretory	5	9
SmLAC36	557	7.08	63050.93	Secretory	5	11
SmLAC37	562	8.84	62954.32	Secretory	6	6
SmLAC38	573	8.54	63309.99	Secretory	8	8
SmLAC39	564	9.45	62407.43	Secretory	7	14
SmLAC40	528	9.21	57151.17	Secretory	7	19
SmLAC41	588	8.91	64864.89	Nuclear	9	15
SmLAC42	549	7.68	62177.20	Mitochondrion	4	7
SmLAC43	612	9.73	68677.13	Secretory	4	9
SmLAC44	575	6.79	64802.50	Secretory	5	12
SmLAC45	572	7.68	64803.49	Secretory	7	9
SmLAC46	530	6.43	59888.48	Secretory	5	11
SmLAC47	523	8.38	59039.55	Secretory	7	8
SmLAC48	562	5.83	63392.48	Secretory	4	8
SmLAC49	551	6.42	61769.98	Secretory	5	12
SmLAC50	560	9.42	61890.78	Secretory	7	11
SmLAC51	556	8.05	63334.11	Mitochondrion	4	5
SmLAC52	565	6.56	63105.85	Secretory	4	9
SmLAC53	571	6.55	63982.28	Secretory	5	11
SmLAC54	564	9.39	62365.35	Secretory	10	10
SmLAC55	572	5.99	62357.02	Secretory	13	11
SmLAC56	579	8.46	66216.14	Secretory	5	5
SmLAC57	507	7.01	55468.35	Secretory	9	10
SmLAC58	562	5.83	63387.5	Secretory	4	8
SmLAC59	570	5.99	62138.75	Secretory	6	10
SmLAC60	557	9.29	60728.01	Secretory	7	12
SmLAC61	506	6.15	56813.46	Secretory	3	9
SmLAC62	528	9.21	57154.22	Secretory	5	19
SmLAC63	630	8.03	70742.12	Secretory	12	8
SmLAC64	506	8.53	55615.62	Secretory	3	14
SmLAC65	587	9.71	65419.69	Secretory	4	15

Generally, most of SmLACs consist of 500–600 amino acids and were probably secreted proteins with a few of them predicted to be located in mitochondria and nuclear. Additionally, variable N- or O-glycosylation sites and phosphorylation sites were predicted to present in all SmLAC proteins, indicating potential posttranslational modifications (Table 1).

### Phylogenetic analysis of LAC proteins

**Figure 1:**
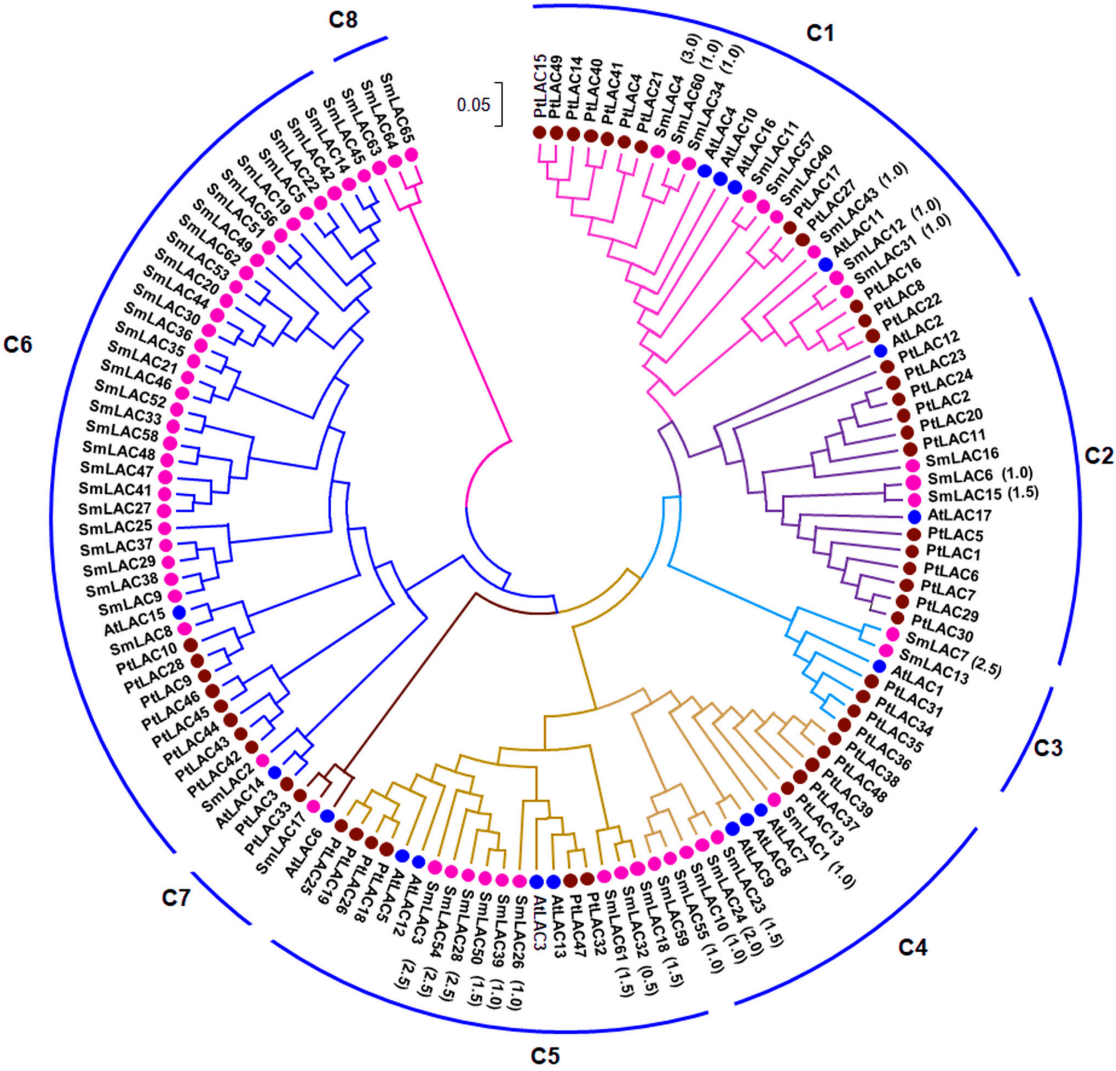
Phylogenetic analysis of SmLAC, AtLAC and PtLAC proteins. The phylogenetic tree of LAC proteins from *S. miltiorrhiza* (red), *Arabidopsis* (blue) and *P. trichocarpa* (green) was constructed using the neighbor-joining method implemented in MEGA7.0 with 1,000 bootstrap replicates.

In order to examine the phylogenetic relationships of SmLACs and LAC proteins from *Arabidopsis* and *P. trichocarpa*, a neighbor-joining (NJ) phylogenetic tree was constructed using MEGA7.0 based on 131 full-length protein sequences of SmLACs, AtLACs and PtLACs (Fig. 1). The constructed tree was highly similar to the trees constructed for AtLACs or for AtLACs and PtLACs (McCaig, Meagher & Dean, 2005; Turlapati et al., 2011; Lu et al., 2013). Based on the tree constructed in this study and those from previous investigations (McCaig, Meagher & Dean, 2005; Turlapati et al., 2011; Lu et al., 2013), we divided the 131 LACs into eight clades (C1–C8), of which seven clades (C1–C7) included proteins from *S. miltiorrhiza*, *Arabidopsis* and *P. trichocarpa*, whereas C8 were specific to *S. miltiorrhiza* (Fig. 1).

Additionally, 42 pairs of SmLACs seem to be paralogous proteins (Table S7). To check the type of selection that acted on genes in the SmLAC family, the ratio between non-synonymous (*Ka*) and synonymous substitutions (*Ks*) was estimated using DnaSP v.5 (Table S7). The results revealed that 13 gene pairs were under strong purifying selection as the *Ka/Ks* ratio was lower than 1.0, while 18 gene pairs were approximately under positive selection with a *Ka/Ks* ratio larger than 1.0. These results showed that different *SmLACs* had undergone different evolutionary forces.

### Conserved domains and gene structures of SmLACs

**Figure 2:**
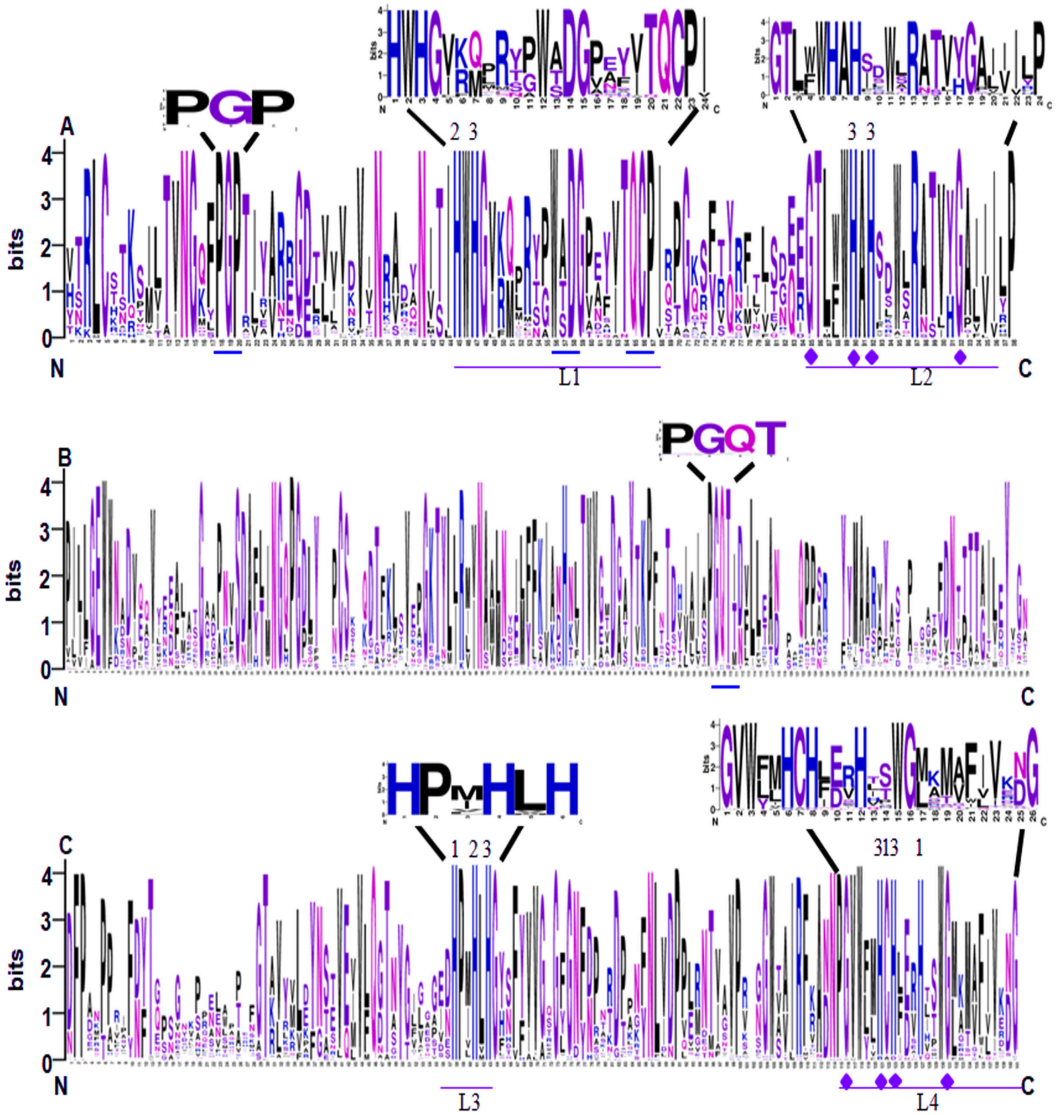
Sequence logo of the conserved domains of SmLACs. (A) Sequence logos of Cu-oxidase_3 domain in SmLACs. (B) Sequence logos of Cu-oxidase domain in SmLACs. (C) Sequence logos of Cu-oxidase_2 domain in SmLAC. The ten conserved histidines and L1–L4 signatures are shown. 1, 2 and 3 indicate the binding sites for T1, T2 and T3 Cu. The blue line indicates the positions of conserved amino acids identified in SmLACs.

Conserved domain analysis showed that all of the 65 identified SmLACs contain three highly conserved Cu-oxidase domains, including Cu-oxidase_3, Cu-oxidase and Cu-oxidase_2 (Fig. 2). Analysis of sequence logos, which reflected the frequency of residue at each position, showed that the distribution of SmLAC residues at the three conserved domains was similar to other plant LACs (McCaig, Meagher & Dean, 2005; Turlapati et al., 2011). This suggests high degree of conservation among plant laccase proteins. In the conserved domains, there are four deeply conserved signature sequences, which were labeled L1 to L4, respectively (Figs. 2A and 2C). It has been shown that these signature sequences are unique for LACs, and ten histidines, one cysteine and one leucine or methionine in the signature sequences are responsible for binding four Cu atoms, including one type-1 (T1) Cu, one type-2 (T2) Cu and two type-3 (T3) Cu (Figs. 2A and 2C) (Turlapati et al., 2011). L1 contains two conserved histidines, one of which binds T2 Cu, whereas the other one binds T3 Cu (Fig. 2A). L2 also includes two conserved histidines, both of which bind T3 Cu (Fig. 2A). L3 contains three histidines, which bind T1, T2 and T3 Cu, respectively (Fig. 2C). L4 includes five conserved Cu-binding residues, three of which bind T1 Cu, whereas the other two bind T3 Cu (Fig. 2C). Further examination of the signature sequences reveals two equivalent motifs, including HWHG in L1 and HLHG in L3 (Figs. 2A and 2C). In addition, there are other three conserved motifs (PGP, WxDG and TQCP) in the Cu-oxidase_3 domain and one conserved PGQT motif in the Cu-oxidase domain (Figs. 2A and 2B).

**Figure 3:**
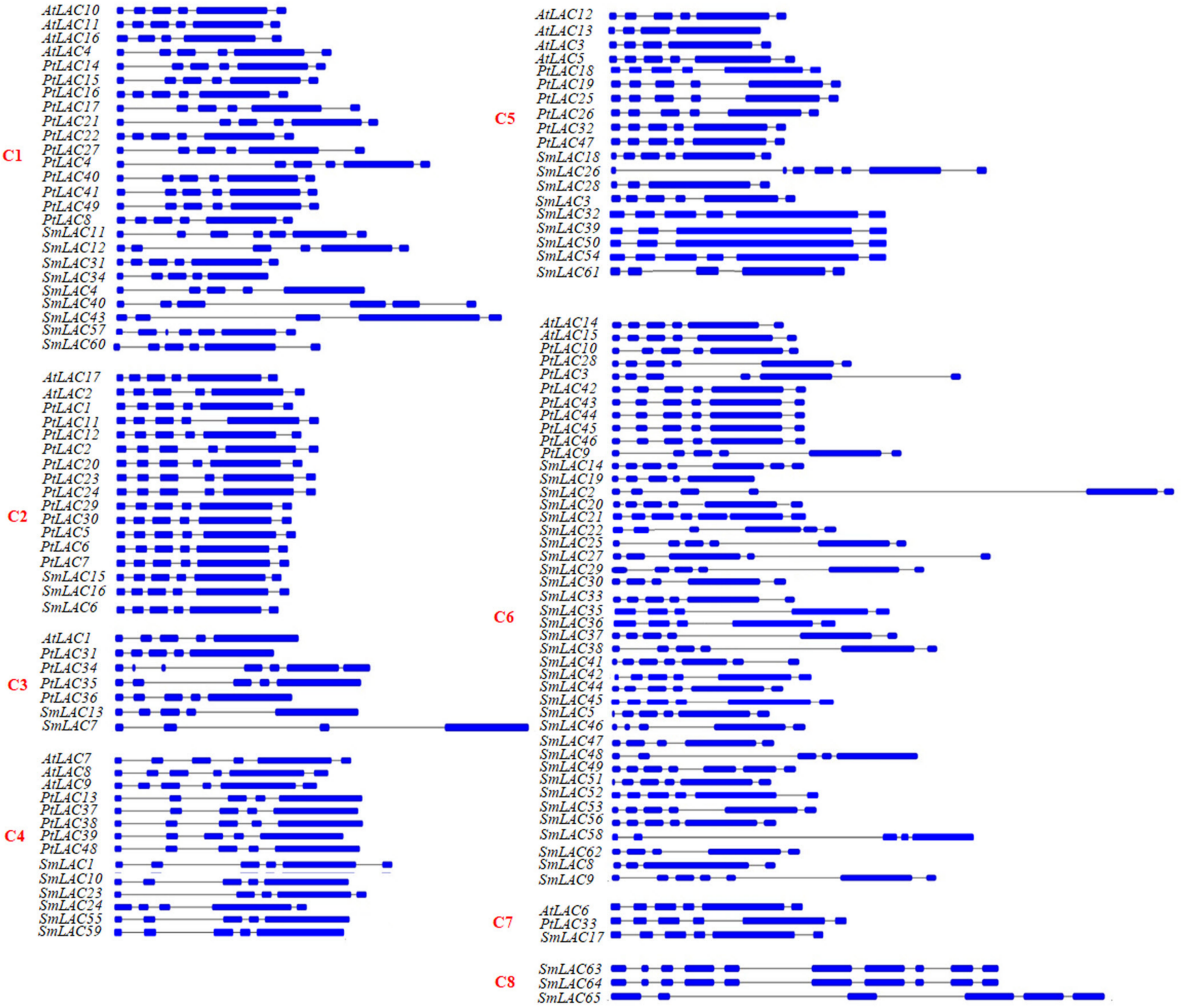
Intron-exon structures of *SmLAC* genes. Blue boxes and lines indicate exons and introns, respectively.

Gene structure analysis showed that the exon number in the coding regions of 65 *S. miltiorrhiza LAC* genes varied between 4 and 10 with the majority (78%) to be 5 or 6 (Fig. 3; Fig. S1). It is consistent with the exon number in *AtLACs* and *PtLACs*, most of which have 5 or 6 exon in the coding regions (Fig. 3; Fig. S1). This suggests the similarity of *S. miltiorrhiza*, *P. trichocarpa* and *A. thaliana LACs* in gene structures. Although the exon number of *SmLACs* is comparable with *AtLACs* and *PtLACs*, several *SmLACs* contain 4 and 10 exons, which represent the greatest difference among the exon numbers of *SmLACs*, *PtLACs* and *AtLACs*. Investigating the structures of genes in each clade showed that C8 genes had intron-exon structures differing from genes in other clades (Fig. 3). This indicates that the ancestors of C8 *SmLACs* genes could be different from other *SmLACs*.

### Diverse *cis* regulatory elements in *SmLAC* promoters

To obtain further insights into the possible expression regulation mode of *SmLACs*, we analyzed the *cis*-acting elements of the 1,500 bp regulatory sequence upstream of the 65 *SmLAC* coding sequences (CDS) using the PlantCARE database (Table S8). The TATA-box elements were found in all *SmLAC* promoter regions. The other *cis*-elements were found that were responsive to hormones or inducers such as methyl jasmonate (MeJA), ethylene, abscisic acid and salicylic acid. Fifty-four members contain abscisic acid (ABA)-responsive elements (ABREs), 41 members have ethylene-responsive elements (EREs), 50 members contain the methyl jasmonate (MeJA)-responsive element (CGTCA motif) and 55 members contain the salicylic acid (SA)-responsive element (TCA element). This analysis revealed a large number of motifs responding to different inductions, suggesting a complex regulation of *SmLAC* genes expression. Besides, we identified some biotic and abiotic stress-related *cis*-acting elements in the upstream regulatory sequences of the *SmLACs*, such as the TC-rich repeat element (related to defence), and low-temperature stress-related (LTR). These results suggest that members of the *SmLAC* family might play roles in the responses to a variety of abiotic and biotic stresses.

### Expression patterns of SmLAC genes in different tissues

**Figure 4:**
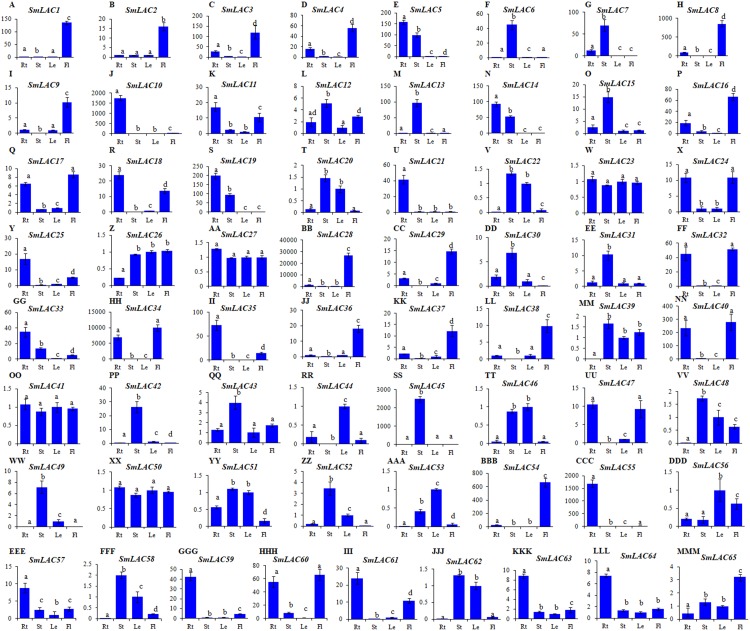
Expression patterns of *SmLACs* (A-MMM) in roots (Rt), stems (St), leaves (Le) and flowers (Fl) of *S. miltiorrhiza*. Expression levels in leaves were arbitrarily set to 1 and the levels in other tissues were given relative to this. Different letters indicate significant differences (ANOVA analysis, *P* < 0.05). Error bars represent standard deviations of mean value from three biological and three technical replicates.

Since gene expression patterns provide clues for deciphering gene functions, we analyzed the level of *SmLAC* transcripts in roots, stems, leaves and flowers of two-year-old, field nursery-grown *S. miltiorrhiza* plants using quantitative real-time RT-PCR. Significant differential expression was observed for 65 of identified *SmLAC* genes (Fig. 4). Among them, 14 (*SmLAC1*, *SmLAC2*, *SmLAC3*, *SmLAC4*, *SmLAC8*, *SmLAC9*, *SmLAC16*, *SmLAC28*, *SmLAC29*, *SmLAC36*, *SmLAC37*, *SmLAC38*, *SmLAC54* and *SmLAC65*) were mainly expressed in flowers, 14 in stems (*SmLAC6*, *SmLAC7*, *SmLAC12*, *SmLAC13*, *SmLAC15*, *SmLAC30*, *SmLAC31*, *SmLAC42*, *SmLAC43 SmLAC45*, *SmLAC48*, *SmLAC49*, *SmLAC52* and *SmLAC58*), 16 in roots (*SmLAC5*, *SmLAC10*, *SmLAC11*, *SmLAC14*, *SmLAC18*, *SmLAC19*, *SmLAC21*, *SmLAC25*, *SmLAC33*, *SmLAC35*, *SmLAC55*, *SmLAC57*, *SmLAC59*, *SmLAC61*, *SmLAC63* and *SmLAC64*), three (*SmLAC44*, *SmLAC53* and *SmLAC56*) in leaves and 14 (*SmLAC17*, *SmLAC20*, *SmLAC22*, *SmLAC24*, *SmLAC26*, *SmLAC32*, *SmLAC34*, *SmLAC39*, *SmLAC40, SmLAC46*, *SmLAC47*, *SmLAC51*, *SmLAC60* and *SmLAC62*) in at least two tissues (Fig. 4). The other four, including *SmLAC23*, *SmLAC27*, *SmLAC41* and *SmLAC50*, were highly expressed in all of the tissues analyzed. The results indicate that *SmLACs* play manifold functions in *S. miltiorrhiza*.

The gene expression of *SmLACs* is highly variable. In fact, folding changes range from units to tens of thousands. Therefore, we verified the amplification efficiency of several PCR primer pairs in low expressed (*SmLAC26* and *SmLAC41*) and highly expressed (*SmLAC28* and *SmLAC34*) *SmLACs*. Polymerase chain reaction efficiency for some primer pairs are listed in Table S9 and gels with expression results of *SmLAC26*, *SmLAC28*, *SmLAC34* and *SmLAC41* are shown in Fig. S2.

### MiRNA-mediated posttranscriptional regulation of SmLAC expression

**Figure 5:**
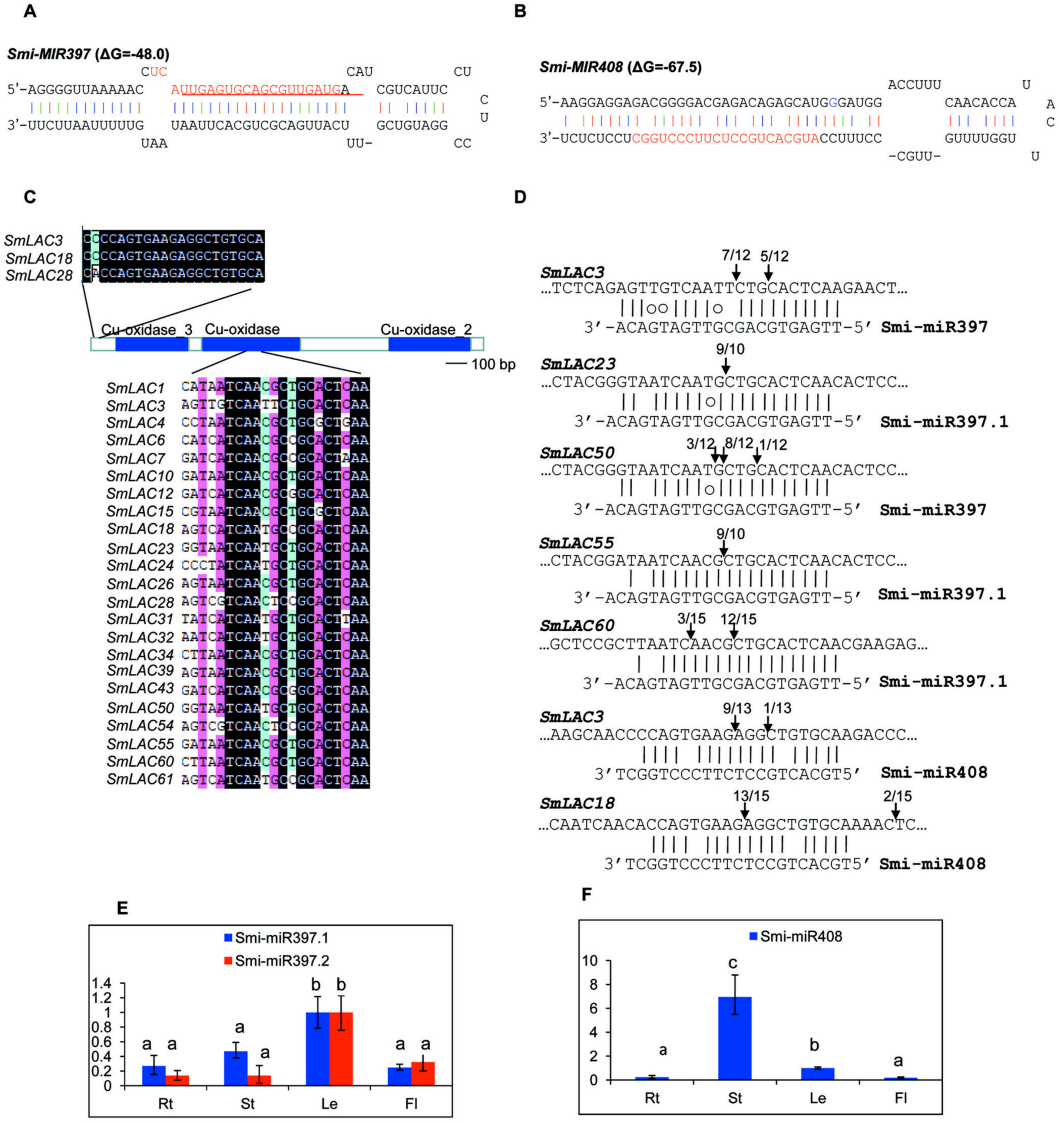
*SmLACs* targeted by Smi-miR397 and Smi-miR408. (A) Hairpin structure of Smi-miR397. Mature miRNA sequences of Smi-miR397.1 and Smi-miR397.2 are indicated by red and red line, respectively. (B) Hairpin structure of Smi-miR408. Mature miRNA sequence of Smi-miR408 is indicated by red. (C) Smi-miR397- and Smi-miR408-targeted *SmLACs*. (D) Experimental validation of Smi-miR397- and Smi-miR408-mediated cleavage of *SmLACs*. Cleavage sites were determined by the modified 5′RNA ligase-mediated RACE method. *SmLAC* sequence of each complementary site from 5′ to 3′ and Smi-miR397/408 sequences from 3′ to 5′ are shown. Smi-miR397/408 complementary sites with the nucleotide positions of *SmLAC* coding region are indicated. Vertical arrows indicate the 5′ termini of miRNA-guided cleavage products, as identified by 5′-RACE, with the frequency of clones shown. (E) and (F) Expression patterns of Smi-miR397.1, Smi-miR397.2 and Smi-miR408 in roots (Rt), stems (St), leaves (Le) and flowers (Fl) of *S*. *miltiorrhiza*, respectively. Expression level of Smi-miR7972 in leaves was arbitrarily set to 1 and the levels of Smi-miR397.1, Smi-miR397.2 and Smi-miR408 were given relative to this. Error bars were indicated by the standard deviations of three biological replicates.

MiRNAs are a class of small non-coding RNAs with 20–24 nucleotides in length. They play vital roles in plant growth, development and stress responses mainly through targeting gene transcripts for cleavage. In *Arabidopsis*, *AtLACs* are posttranscriptionally regulated by miR397, miR408 and miR857 (Abdel-Ghany & Pilon, 2008). In order to examine whether *SmLACs* are regulated by miRNAs, we searched *S. miltiorrhiza* small RNAs (Xu et al., 2014) potentially targeting *SmLACs* for cleavage using psRNATarget with default parameters (Dai & Zhao, 2011). Using penalty score cutoff threshold 3 for mismatched patterns in the miRNA/target duplexes as the criterion, we identified a total of 34 small RNAs. We mapped the retrieved small RNA sequences to the assembly of *S*. *miltiorrhiza* genome (Xu et al., 2016). Genomic sequences surrounding the matched regions were then analyzed for secondary structures using mfold (Zuker, 2003). Finally, two stem-loop structures satisfying the criteria of miRNA precursors (Meyers et al., 2008) were identified. They are *Smi-MIR397* (Fig. 5A) and *Smi-MIR408* (Fig. 5B). *Smi-MIR397* generated two overlapped mature miRNAs, termed Smi-miR397.1 and Smi-miR397.2, respectively. *Smi-MIR408* generated one mature miRNA, termed Smi-miR408. Computational prediction showed that 23 of the 65 identified *SmLACs* had near-perfect complementary region to Smi-miR397.1, Smi-miR397.2 and Smi-miR408 (Fig. 5C). Among them, 11 *SmLACs* are targets of Smi-miR397.1, and 9 are common targets of Smi-miR397.1 and Smi-miR397.2. Two, including *SmLAC3* and *SmLAC18* are common targets of Smi-miR397.1, Smi-miR397.2 and Smi-miR408, and *SmLAC28* is common targets of Smi-miR397.1 and Smi-miR408. The target site of Smi-miR408 locates at the nonconserved 5′ terminal region, whereas the site of Smi-miR397 locates in the region encoding the conserved Cu-oxidase domain.

Using the poly(A) adaptor RT-PCR method, we analyzed the expression of Smi-miR397.1, Smi-miR397.2 and Smi-miR408 in roots, stems, leaves and flowers of 2-year-old, field-grown *S. miltiorrhiza* Bunge (line 993). Smi-miR397 exhibited higher expression level in leaves compared with the level in roots, stems and flowers (Fig. 5E). Smi-miR408 showed the highest expression in stems. Its expression in roots and flowers is similar (Fig. 5F).

**Figure 6:**
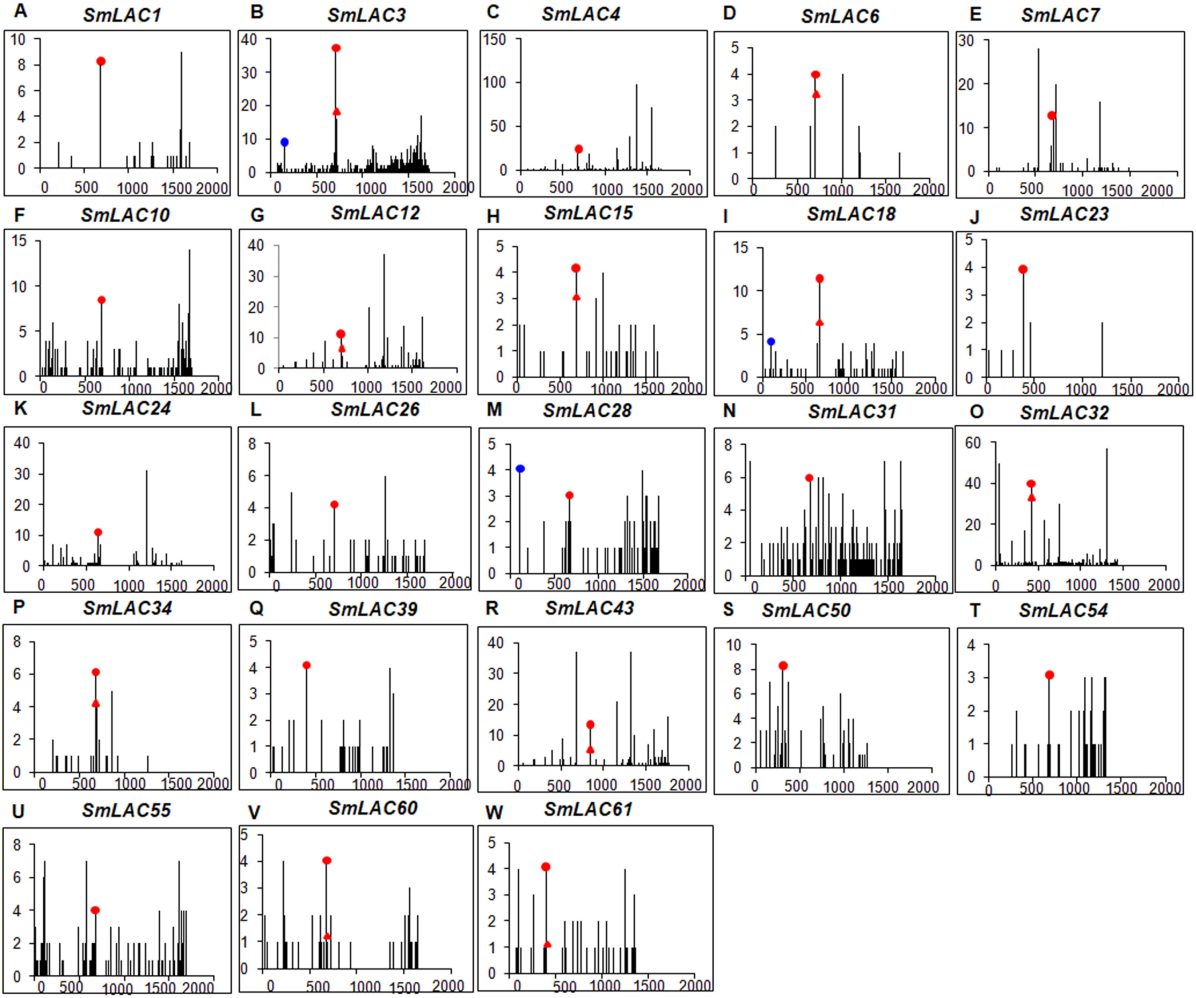
Degradome analysis of Smi-miR397- and Smi-miR408-directed cleavage of *SmLACs*. (A–W) X-axis represents the nucleotide position in SmLAC coding region. Y-axis represents the number of reads of cleaved transcripts detected in the degradome database. Each black line represents the number of degradome fragments mapped to the position. The red spots and red triangles indicate that the products are resulted from Smi-miR397.1 and Smi-miR397.2-directed cleavage, respectively. The blue spots indicate that the products are resulted from Smi-miR408-directed cleavage.

Plant miRNA-mediated cleavage of transcripts usually locates at a site corresponding to the tenth miRNA nucleotide from the 5′ end. To validate the cleavage of Smi-miR397 and Smi-miR408 on *SmLACs*, we analyzed high-throughput *S. miltiorrhiza* degradome data (Xu et al., 2014). The results confirmed that the 23 *SmLACs* were targets of Smi-miR397 and Smi-miR408 (Fig. 6). Furthermore, we performed the modified 5′ RNA ligase-mediated RACE (5′RLM RACE) for mapping of cleavage sites. The experiments were carried out on mRNA isolated from roots, stems, leaves and flowers of *S. miltiorrhiza*. The 5′RLM RACE products revealed that six Sm*LACs*, including *SmLAC3*, *SmLAC18*, *SmLAC23*, *SmLAC50*, *SmLAC55* and *SmLAC60*, were indeed cleaved by Smi-miR397 and/or Smi-miR408 in vivo (Fig. 5D). Further investigating the cleavage sites of Smi-miR397 showed that *SmLAC3* and *SmLAC50* were cleaved by both Smi-miR397.1 and Smi-miR397.2.

The 23 miRNA-targeted *SmLACs* distribute in five clades of the phylogenetic tree (Fig. 1). *SmLACs* targeted by Smi-miR397 distribute in C1, C2, C3, C4 and C5, and those targeted by Smi-miR408 belong to C5. No miRNA targets were found in C6, C7 and C8. It is consistent with the fact that LACs in a clade show relatively high sequence similarity and usually play redundant functions.

### Putative functions of *SmLACs*

All the identified proteins were subjected to GO annotations to understand the biological functions. The GO database contains three ontologies: biological process, cellular component and molecular function. Thirty-five SmLAC proteins were categorized into the three ontologies (Fig. S3; Table S10). Among the 35 SmLACs, 3 SmLACs (SmLAC63, SmLAC64 and SmLAC65), 13 SmLACs and 34 SmLACs were annotated in the cellular component, molecular function and biological process categories, respectively (Table S10). Further analysis of GO annotations showed that *SmLAC* genes involved in the biosynthesis of secondary metabolites, such as lignin biosynthetic process, phenylpropanoid metabolic process, plant-type secondary cell-wall biogenesis and proanthocyanidin biosynthetic process (Fig. S3; Table S10).

Since functional redundancy of plant *LACs*, it is difficult to accurately uncover the role of single *SmLAC* in plants. However, clues can be acquired from phylogenetic analysis and expression profiling. *AtLAC2* that belong to C2 was widely expressed in roots, stems, leaves and flowers with the level in stems relatively high (Turlapati et al., 2011). It was down-regulated under heat, drought, genotoxic (bleomycin and mitomycin), brassinolide, IAA, salicylic acid and zeatin treatments (Turlapati et al., 2011). Knockout mutant of *AtLAC2* exhibited reduced root elongation under PEG-induced dehydration conditions (Cai et al., 2006). Although the function of *AtLAC2* has not been fully elucidated, current evidence indicates that it is involved in the development of stems and roots and in plant response to various stresses.

Laccases are associated with monolignols or flavonoid metabolites in plants (Table S11). The *Arabidopsis AtLAC* member of C1 and C2, *AtLAC17*, *AtLAC4*, *AtLAC10* and *AtLAC11* were widely expressed in roots, stems, leaves and flowers with the highest expression level in stems (Turlapati et al., 2011). Double mutation of *AtLAC4* and *AtLAC17* resulted in significant reduction of lignin content (Berthet et al., 2011). Triple mutation of *AtLAC4*, *AtLAC11* and *AtLAC17* resulted in extremely low of lignin content in roots (Zhao et al., 2013). Consistently, in Ptr-miR397a-overexpressed *P. trichocarpa* trangenics, the reduction of lignin content in stem xylem was accompanied by significantly decreased expression of a subset of *PtLAC* genes, the majority of which, such as *PtLAC1*, *PtLAC2*, *PtLAC14*, *PtLAC15*, *PtLAC20*, *PtLAC23*, *PtLAC24*, *PtLAC40*, *PtLAC41* and *PtLAC49*, are members of C1 and C2 (Lu et al., 2013). This suggests that C1 and C2 *LAC* members are probably involved in lignin biosynthesis. A total of 12 *SmLACs* are included in C1 and C2. Five of them, including *SmLAC6*, *SmLAC12*, *SmLAC15, SmLAC31* and *SmLAC43* were predominantly expressed in stems (Fig. 4), and all of them are targets of Smi-miR397 (Fig. 5C). This indicates the putative roles of these five *SmLACs* in oxidative lignin polymerization in *S. miltiorrhiza* and suggests the conserved regulatory mechanism of miR397-LAC module in oxidative polymerization in *S. miltiorrhiza* and *Arabidopsis*.

It has been shown that *AtLAC15* is involved in oxidative polymerization of flavonoids (Pourcel et al., 2005). The seeds of *lac15-1* and *lac15-2* mutants showed yellow or pale color, resembling a *transparent testa* phenotype (Cai et al., 2006). *SmLAC8* showed very close phylogenetic relationship with *AtLAC15* (Fig. 1) and were predominantly expressed in flowers (Fig. 4). This indicates the role of *SmLAC8* in the polymerization of flavonoids. In addition, *SmLACs* in C8 could be involved in the biosynthesis of lineage-specific compounds, such as salvianolic acids, since no *Arabidopsis* and *P. trichocarpa* homologs were found for these *SmLACs* (Fig. 1). Further functional characterization of *SmLACs* through genetic transformation and enzyme activity analysis will definitely shed lights on the mechanisms of *SmLAC*-mediated polymerization of phenolic compounds.

## Discussion

Laccases widely exist in various plant species, such as *Arabidopsis thaliana* (McCaig, Meagher & Dean, 2005; Turlapati et al., 2011), rice (Liu et al., 2017), tobacco (Richardson & McDougall, 1997), ryegrass (Gavnholt, Larsen & Rasmussen, 2002), cotton (Balasubramanian et al., 2016), poplar (LaFayette, Eriksson & Dean, 1999; Lu et al., 2013), sycamore maple (Sterjiades, Dean & Eriksson, 1992) and pear (Cheng et al., 2019). Here, we identified and characterized 65 LAC genes in *S. miltiorrhiza*. They all contain the typical characteristics of three conserved Cu-oxidase domains and SmLACs are mostly secretory proteins. Integrative analysis of gene structures, sequence features, conserved domains and phylogenetic relationships showed that *SmLAC* genes could be divided into eight clades. Previous results showed that 17 AtLACs and 49 PtLACs were clustered into six clades (McCaig, Meagher & Dean, 2005; Turlapati et al., 2011; Lu et al., 2013). Comparison of our results with previous studies showed that, among the seven clades with SmLAC, AtLAC and PtLAC proteins, six clades (C1–C6) were consistent, whereas C7 was an extra. C7 consists of three proteins, including AtLAC6, PtLAC33 and SmLAC17. AtLAC6 and PtLAC33 belonged to C6 in the phylogenetic trees constructed previously (Lu et al., 2013), although it showed relative far relationships with other members in the clade. Adding SmLACs to the tree resulted in separation of AtLAC6 and PtLAC33 from C6. Similar results were observed in a tree constructed for AtLACs and *Citrus sinensis* CsLACs (Xu et al., 2019). This suggests that the relationships between C6 and C7 members are indeed relatively far. C8 was specific to *S. miltiorrhiza*, indicated that *SmLACs* in C8 could be involved in the biosynthesis of lineage-specific compounds.

It has been reported that these three *Arabidopsis* laccase genes were up-regulated by stress. *AtLAC4*, *AtLAC10* and *AtLAC11* that belong to C1 and *AtLAC2* and *AtLAC17* that belong to C2, are highly expressed in stems (Fig. 1) (Turlapati et al., 2011). Among them, AtLAC2, AtLAC4 and AtLAC17 are targets of Ath-miR397 (Abdel-Ghany & Pilon, 2008). *AtLAC2* was widely expressed in roots, stems, leaves and flowers with the level in stems relatively high (Turlapati et al., 2011). It was down-regulated under heat, drought, genotoxic (bleomycin and mitomycin), brassinolide, IAA, salicylic acid and zeatin treatments (Turlapati et al., 2011). Knockout mutant of *AtLAC2* exhibited reduced root elongation under PEG-induced dehydration conditions (Cai et al., 2006). Although the function of *AtLAC2* has not been fully elucidated, current evidence indicates that it is involved in the development of stems and roots and in plant response to various stresses.

MiRNAs have been demonstrated to play important roles in negatively regulating the expression of laccase genes by degradation of target mRNAs in several plants, such as miR408, miR397 and miR857 in *Arabidopsis* regulating seven laccase genes, ptr-miR397a in *P. trichocarpa* regulating seventeen laccase genes, and Os-miR397 in *Oryza sativa* regulating OsLAC gene (Abdel-Ghany & Pilon, 2008; Lu et al., 2013; Zhang et al., 2013). Our results suggested that 23 *SmLACs* were predicted to be Sm-miR397 targets. All these SmLACs were clustered within C1, C2, C3, C4 and C5 in the phylogenetic tree. This distribution indicated the role of Smi-miR397 in controlling cell-wall lignin biosynthesis and stress responses.

## Conclusions

A total of 65 *SmLAC* genes were identified in *S. miltiorrhiza*. Genes and the deduced proteins were characterized through a comprehensive approach, including phylogenetic tree construction, laccase domain characterization, gene structure analysis and gene expression profiling. The majority of clades contained members from *S. miltiorrhiza*, *A. thaliana* and *P. trichocarpa*, which indicated that the function of most LACs was conserved among plant species. However, C8 was specific to *S. miltiorrhiza*. This indicates that the members of the clade play unique functions in *S. miltiorrhiza*. The Cu_oxidase domains are highly conserved in plant LACs. Gene structures of most *LACs* from *S. miltiorrhiza*, *Arabidopsis* and *P. trichocarpa* were highly conserved. However, *SmLACs* encoding C8 proteins were distinct. Although the precise functions of most plant LACs remain unknown, phylogenetic and expression analyses showed that LACs could play conserved and divergent roles in polymerization of phenolic compounds. Posttranscriptional analysis revealed that Smi-miR397 and Smi-miR408 were involved in regulating a subset of *S. miltiorrhiza LAC* genes.

## Supplemental Information

10.7717/peerj.7605/supp-1 Supplemental Information 1 Exon number of *LAC* in *S. miltiorrhiza*, *P. trichocarpa* and *Arabidopsis*. A, B and C:exon number in the coding region of 65 *S. miltiorrhiza* (A), 49 *P. trichocarpa* (B) and 17 *Arabidopsis LAC* genes (C)

10.7717/peerj.7605/supp-2 Supplemental Information 2 Expression patterns of *SmLACs* in roots (Rt), stems (St), leaves (Le) and flowers (Fl) of *S. miltiorrhiza*.

10.7717/peerj.7605/supp-3 Supplemental Information 3 Distribution of SmLACs proteins classified into different GO terms.

10.7717/peerj.7605/supp-4 Supplemental Information 4 Primers used for 5′-RACE of *SmLACs*.

10.7717/peerj.7605/supp-5 Supplemental Information 5 Primers used for 3′-RACE of *SmLACs*.

10.7717/peerj.7605/supp-6 Supplemental Information 6 Primers used for the PCR amplification of coding sequences of *SmLACs*. CDS sequences of LACs in *S. miltiorrhiza*

10.7717/peerj.7605/supp-7 Supplemental Information 7 Primers used for quantitative real-time RT-PCR of *SmLACs*.

10.7717/peerj.7605/supp-8 Supplemental Information 8 Primers used for analysis of Sm-miR397-directed cleavage of *SmLACs*.

10.7717/peerj.7605/supp-9 Supplemental Information 9 Primers used for analysis of Sm-miR408-directed cleavage of *SmLACs*.

10.7717/peerj.7605/supp-10 Supplemental Information 10 Ka/Ks analysis for *SmLAC* paralogous genes from *S. miltiorrhiza*.

10.7717/peerj.7605/supp-11 Supplemental Information 11 Numbers of *cis*-elements in promoter region of *SmLAC*s.

10.7717/peerj.7605/supp-12 Supplemental Information 12 PCR efficiency of each *SmLAC* qRT-PCR primer.

10.7717/peerj.7605/supp-13 Supplemental Information 13 Gene Ontology terms of SmLACs.

10.7717/peerj.7605/supp-14 Supplemental Information 14 Summary in secondary metabolism of LAC.

10.7717/peerj.7605/supp-15 Supplemental Information 15 Open reading frame (ORF) sequences of LACs in *S. miltiorrhiza*.

10.7717/peerj.7605/supp-16 Supplemental Information 16 Expression patterns of*SmLACs*in roots (Rt), stems (St), leaves (Le) and flowers (Fl) of *S. miltiorrhiza*.

## Additional Information and Declarations

### Data Availability

The following information was supplied regarding data availability: The raw data are available in the Supplemental Files.
