# Supplementary figures and images for: Analysis of the laccase gene family and miR397-/miR408-mediated posttranscriptional regulation in Salvia miltiorrhiza
Source: PeerJ. 2019 Aug 29;7:e7605. doi: 10.7717/peerj.7605 (PMC6717658; doi:10.7717/peerj.7605)

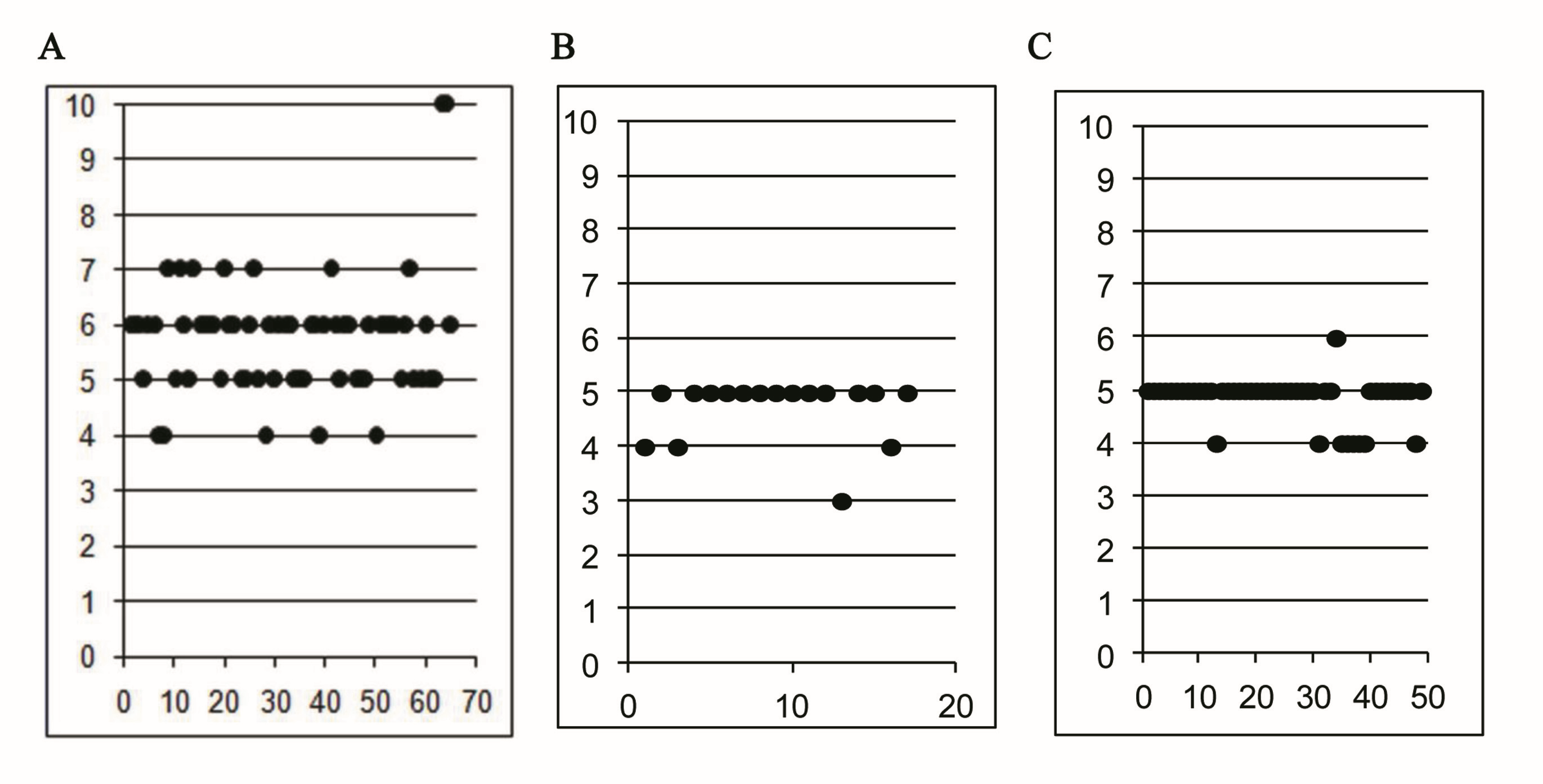

Supplement: Supplemental Information 1 — A, B and C:exon number in the coding region of 65 S. miltiorrhiza (A), 49 P. trichocarpa (B) and 17 Arabidopsis LAC genes (C) [file peerj-07-7605-s001.png]

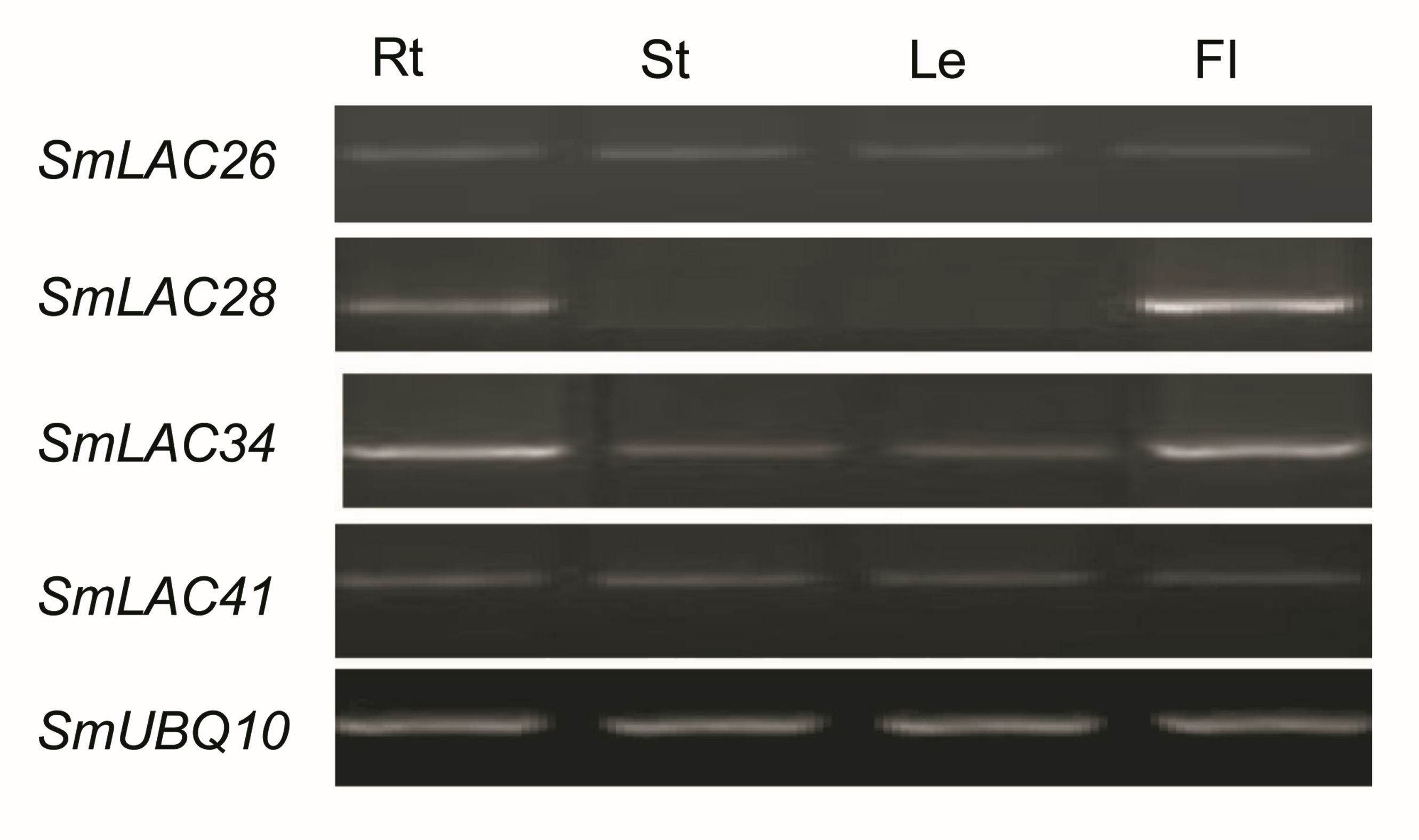

Supplement: Supplemental Information 2 [file peerj-07-7605-s002.png]

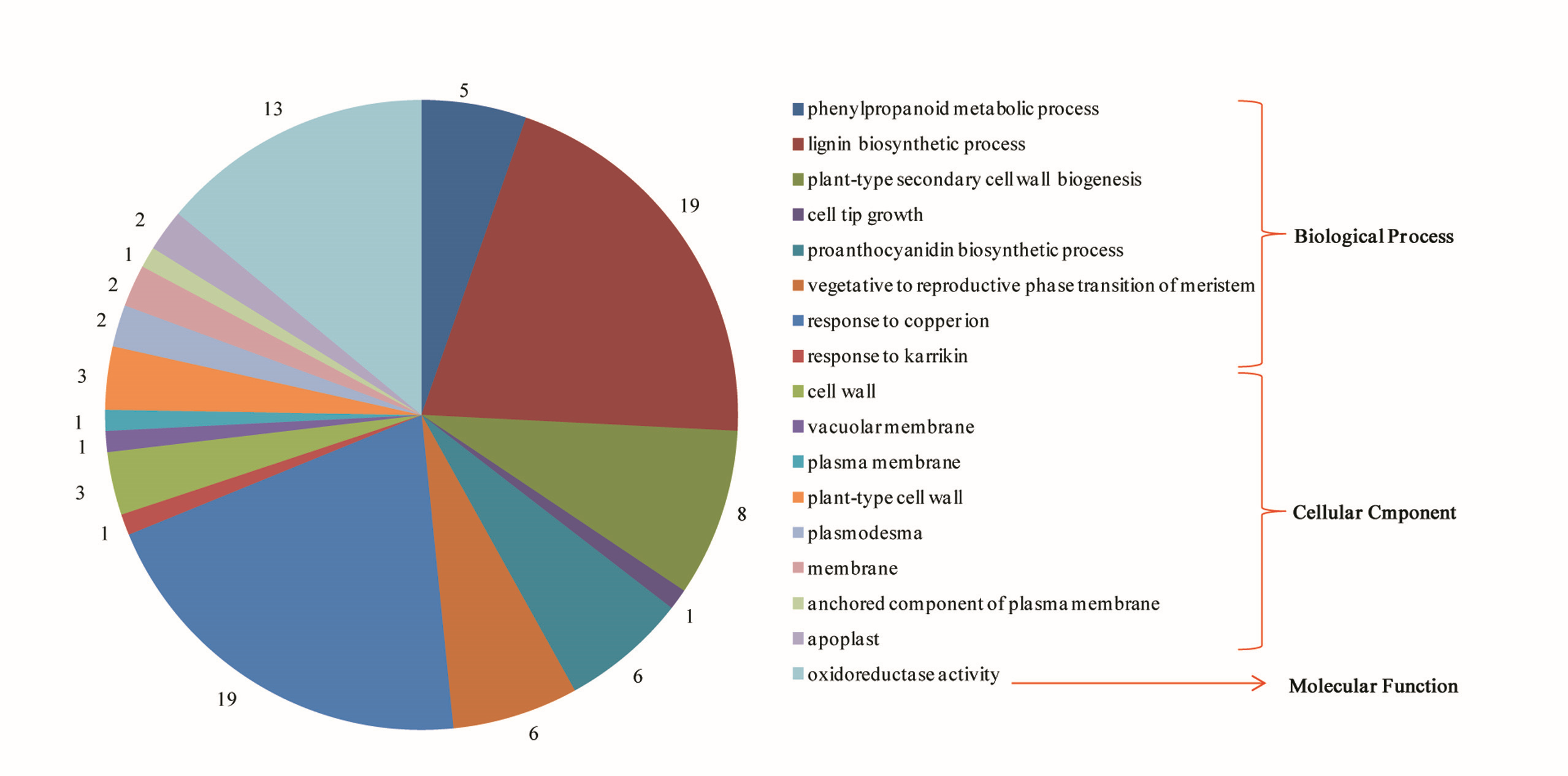

Supplement: Supplemental Information 3 [file peerj-07-7605-s003.png]
