# Supplementary material for: Analysis of the laccase gene family and miR397-/miR408-mediated posttranscriptional regulation in Salvia miltiorrhiza
Source: PeerJ. 2019 Aug 29;7:e7605. doi: 10.7717/peerj.7605 (PMC6717658; doi:10.7717/peerj.7605)
Supplement: Supplemental Information 4 [file peerj-07-7605-s004.docx]

**Table S1** Primers used for 5’-RACE of *SmLACs*.

| Gene name | Primer sequence (5’-3’) |
| --- | --- |
| *SmLAC4* | Nesting: GCTACGTAGGCACTCGCCGCC |
|  | Nested: GTAGGCCGCGTCGACGGCTACCACC |
| *SmLAC7* | Nesting: GAACTTTCCGTCCACGGTTTGCGG |
|  | Nested: CGTCGTTGAGCGCAGCGTTGATC |
| *SmLAC10* | Nesting: GTACGCGGTCACCGTGTTCGTGTCG |
|  | Nested: CCGACCATGATGACTCTGGTGGAGAAG |
| *SmLAC15* | Nesting: AGGTTGTGGTTGGCGATTTTGAAGAAC |
|  | Nested: GATCCGCTTTGAAGGCCTTCCCTC |
| *SmLAC17* | Nesting: GCCATGTAGTAGCGCGCGGGTGGC |
|  | Nested: ACTGAGATTTTGGTTGTGCCTTGGC |
| *SmLAC19* | Nesting: GAAGAACATTATGTTGTCCATCACCGCG |
|  | Nested: GGGAAGATGTGCATGAGGCTTCGGAAAC |
| *SmLAC20* | Nesting: GGGAAAGGATAAGTGTGTGTCTTGGGC |
|  | Nested: GAACTCGTTGTCGAAAGCTTTTGCCGG |
| *SmLAC21* | Nesting: TGCGTGTAGTTGAAGACGAAGCCCG |
|  | Nested: CGCTGAGTCGCCGGTAGTAGGCCTG |
| *SmLAC23* | Nesting: GAGCATGAGGACGGAGGTGGAGAAGGG |
|  | Nested: CAAGAAGAAGGGGAGTTTCACGGTGG |
| *SmLAC26* | Nesting: GGTGACGTTGTGGCCGCCGATTTTGAAGAAC |
|  | Nested: GAGCAAGGGTACAAGTAACCTGGTTGACC |
| *SmLAC28* | Nesting: CTATGGTCTGGCCGGGGGCGATGG |
|  | Nested: GAAGTACATAATGTAGTTCATCATTGC |
| *SmLAC31* | Nesting: GCAGCATTGATGATACGCAGCAGGTACG |
|  | Nested: GTGGCCCTGGCTTCCCATTGATTGTGTG |
| *SmLAC32* | Nesting: GTCGGGGAGTTGGCAAGGGCGCCGG |
|  | Nested: CGACCACCGTCAGTTTGTGGCCGGCG |
| *SmLAC34* | Nesting: CAGAACATTGGTGGTCTGCCCTGGTCCG |
|  | Nested: GTTGTAGGCGGCGTCGTTCCCGACCACGG |
| *SmLAC37* | Nesting: CGATCGCGATGTGATCGGTGTTCAGCGGC |
|  | Nested: GGTAAGTCTTGCCCGGCTGCACACTC |
