# Supplementary material for: Analysis of the laccase gene family and miR397-/miR408-mediated posttranscriptional regulation in Salvia miltiorrhiza
Source: PeerJ. 2019 Aug 29;7:e7605. doi: 10.7717/peerj.7605 (PMC6717658; doi:10.7717/peerj.7605)
Supplement: Supplemental Information 5 [file peerj-07-7605-s005.docx]

**Table S2** Primers used for 3’-RACE of *SmLACs*.

| Gene name | Primer sequence (5’-3’) |
| --- | --- |
| *SmLAC21* | Nesting: CGGGCTTCGTCTTCAACTACACGCA |
|  | Nested: CAGGCCTACTACCGGCGACTCAGCG |
| *SmLAC30* | Nesting: CGTCAAAGTCGTCAACTACAAGCTC |
|  | Nested: GTCAACTCTGCACTCAACCAAGAACTC |
| *SmLAC32* | Nesting: CCGGCGCCCTTGCCAACTCCCCGAC |
|  | Nested: GCCGCAAAAGATCGACCACTCCCTG |
