# Supplementary material for: Analysis of the laccase gene family and miR397-/miR408-mediated posttranscriptional regulation in Salvia miltiorrhiza
Source: PeerJ. 2019 Aug 29;7:e7605. doi: 10.7717/peerj.7605 (PMC6717658; doi:10.7717/peerj.7605)
Supplement: Supplemental Information 6 — CDS sequences of LACs in S. miltiorrhiza [file peerj-07-7605-s006.docx]

**Table S3** Primers used for the PCR-amplification of coding sequences of *SmLACs*

| Gene name | Primer sequence (5’-3’) |
| --- | --- |
| *SmLAC4* | Forward: ATGGATTCTTGGATGATTAGGGCTTTC |
|  | Reverse: TTATGAGCATGTGGGTAAATCTTGAGGC |
| *SmLAC7* | Forward: ATGAGAGGGAACGAAGCCGAGCAAG |
|  | Reverse: TTAGACTGAGCATGGAGGTAGATCATTAG |
| *SmLAC10* | Forward: ATGCGTCGTTCCTTGGTATTTCTAG |
|  | Reverse: CTAGCATTGTGGAAGATCCGCAGGAG |
| *SmLAC15* | Forward: ATGGGTTTGCAAATTTCTATAGC |
|  | Reverse: TCAACATTTAGGAAGATCGGAAGGC |
| *SmLAC17* | Forward: ATGGCAGTCACAACCACTGCCCTCG |
|  | Reverse: TCAGCTGGAGGGTGGGGGAGGGTCTCGAG |
| *SmLAC19* | Forward: ATGTTTTCGACTAGGAAGGTTTTCG |
|  | Reverse: TTAAATCCTAAACAATAATTGTTGAGATG |
| *SmLAC20* | Forward: ATGTTTTATTATCGGAAGCTTAAGG |
|  | Reverse: CTAAATTGTTAAGGCTGCAGGGGGC |
| *SmLAC21* | Forward: ATGCTAACTGAATATTTTACATGTGTGC |
|  | Nested: TCAAATCCTAAATACTAATGCTTGAGGG |
| *SmLAC23* | Forward: ATGTACGTCGGGGTGGTGAGGAGCG |
|  | Reverse: CTAGCATTTGGGAAGATCTGCAGGTGG |
| *SmLAC26* | Forward: ATGAAGGATGCGGAGGTTGGTGATAG |
|  | Reverse: TCAGCATTGTGGGAGATCAGCCGGCGG |
| *SmLAC28* | Forward: ATGAGCTCTTTGTCTGCGTTGGCATTG |
|  | Reverse: TCAACAAAGGGGAAGATCTGGTGGAGG |
| *SmLAC30* | Forward: ATGCTAACGGTAAACGGGCAGTTCCCAG |
|  | Reverse: CTAAATTGTTGAGGCTGCAGGGGGCGG |
| *SmLAC31* | Forward: ATGGGGAGAACACATGCTTTTGGTG |
|  | Reverse: TTAACAATGTGGAAGATCTTTAGGTGG |
| *SmLAC32* | Forward: ATGCGAACGCCTTGGGCTGACGGGCC |
|  | Reverse: CTAACAAGGAGGGAGATCAGGTGGAG |
| *SmLAC34* | Forward: ATGGAAGTCTGGGTTCGTGTTTTGATC |
|  | Reverse: CTACCGCGAAGAAATTGAATCCGTGCAG |
| *SmLAC37* | Forward: ATGGGAACTACCAAGACGTTGATCTTG |
|  | Reverse: TCAGCATCTTGGGAAATCTGGAGGTGGAG |
