# Supplementary material for: Analysis of the laccase gene family and miR397-/miR408-mediated posttranscriptional regulation in Salvia miltiorrhiza
Source: PeerJ. 2019 Aug 29;7:e7605. doi: 10.7717/peerj.7605 (PMC6717658; doi:10.7717/peerj.7605)
Supplement: Supplemental Information 7 [file peerj-07-7605-s007.docx]

**Table S4** Primers used for quantitative real-time RT-PCR of *SmLACs*

| Gene name | Primer sequence (5’-3’) |
| --- | --- |
| *SmLAC1* | Forward: TAACAGTGGTGCACGGAAAG |
|  | Reverse: CGCAACTACGGTGAGGTTATG |
| *SmLAC2* | Forward: GCATGGGTATGGGTTCTATGT |
|  | Reverse: CGGAGGGTCATCGAGATTATAC |
| *SmLAC3* | Forward: GCTCCAGCAAAGACACAGTA |
|  | Reverse: GTGATTGGCCACTTTGAAGAAG |
| *SmLAC4* | Forward: TAGAGCGGGCAAATCATACC |
|  | Reverse: TCCACGACGGTGAAATTGT |
| *SmLAC5* | Forward: GGAAGTTGAGATCGTGCTTCA |
|  | Reverse: CCCGAATCCAGTTCCGATAAC |
| *SmLAC6* | Forward: TACCTCCTCCGCATCATCAA |
|  | Reverse: TGGAAGGGCTTGACGTAGA |
| *SmLAC7* | Forward: ACCCTTGTTCCACCAAAGATAC |
|  | Reverse: CGAAGAAGAGCTCATCGTTTAGT |
| *SmLAC8* | Forward: GCTGAGGTACAACTCCAACAT |
|  | Reverse: CCCAACCAACAACGAAGAAAC |
| *SmLAC9* | Forward: TTGGACCGCGATTAGATTCC |
|  | Reverse: GTTCGTGCCTTTCCCATTTC |
| *SmLAC10* | Forward: ACACGGTTAGGTTCACGTTAG |
|  | Reverse: CGATGGCTACCACTGTGAAT |
| *SmLAC11* | Forward: TGTACAGACTGGCGTACAATTC |
|  | Reverse: TGGATCCGTGCAAATGGATAG |
| *SmLAC12* | Forward: CCTACCTTCTCAGGATCATCAAC |
|  | Reverse: GATCTCCACAACCGTCATGT |
| *SmLAC13* | Forward: TACACAGCCGATTTCATCCC |
|  | Reverse: AACTACCTCGATGTTGGAGTTG |
| *SmLAC14* | Forward: CGACTTCACGCAAGGCTATAA |
|  | Reverse: TGAAGCACGATCTCAACTTCC |
| *SmLAC15* | Forward: CAACTGCTCCACCAAAGATACA |
|  | Reverse: TTGGCGATGCTGAAGAAGAG |
| *SmLAC16* | Forward: GGAGTCTACACCACAGATTTCC |
|  | Reverse: CGAGTTCCATTGCTCACTACA |
| *SmLAC17* | Forward: CAGGGATAAAGCTGGCTACTTC |
|  | Reverse: TCATCGCGTTGGTGTCATTAG |
| *SmLAC18* | Forward: GAGACACTACTCCTGAGAGTCA |
|  | Reverse: CTGGTGGAGAAGGGTTTGTT |
| *SmLAC19* | Forward: GGTTCGGATGCTGGAGTATAAC |
|  | Reverse: CCGACCACATAGAAGCTGTATC |
| *SmLAC20* | Forward: GGTTGATGCACTGCCATTTC |
|  | Reverse: CGGAGGCAACATCTTCTCAT |
| *SmLAC21* | Forward: GGGAATCGATCATCCCATACAT |
|  | Reverse: CGGTGGGTCAACGAGATTATAG |
| *SmLAC22* | Forward: CGACTTCACGGAAGGTTATAGG |
|  | Reverse: ATTCCATCTCCAACGCCATAG |
| *SmLAC23* | Forward: GGTTAGGTTCACGTTGGTACAT |
|  | Reverse: GAATTTGTGGTTGGCGATCTTG |
| *SmLAC24* | Forward: ACGTTTAAGCTGAGGGTGAAG |
|  | Reverse: GAGCGTGTGGTTGGAGATT |
| *SmLAC25* | Forward: CTCCATGGGTACAGCTTCTATG |
|  | Reverse: CAGAGGAGGATCGACAAGATTATAG |
| *SmLAC26* | Forward: GACACGGTGATGCTAAGAGTAA |
|  | Reverse: GGTGAAGGGCCTGTTGTAG |
| *SmLAC27* | Forward: GTGTTTCAAGCGACCAACATC |
|  | Reverse: TTCCGTCGAAGTTTCCTAACC |
| *SmLAC28* | Forward: CTCCGCCAAACACACTCTTAT |
|  | Reverse: TGGTTGGCGACTTTGAAGAA |
| *SmLAC29* | Forward: GATGCATTTGCACGGGTATAG |
|  | Reverse: ATGGCGGATCGACAAGATTA |
| *SmLAC30* | Forward: TTCAGCAACAGGCTCAGAAG |
|  | Reverse: CCACGTATTGATGGAGAGAGTG |
| *SmLAC31* | Forward: GTACCTGCTGCGTATCATCAA |
|  | Reverse: GTACACTGCATCCACCTCAA |
| *SmLAC32* | Forward: GCTTCTCTTGAGAATCATCAATGC |
|  | Reverse: GTCCGATCATGACCACTCTG |
| *SmLAC33* | Forward: CTCAGAAGTGGAGATCGTGTTT |
|  | Reverse: CCGACAACGTAGAAGCTGTATC |
| *SmLAC34* | Forward: CTTCACCCTCTCCATCAATCTC |
|  | Reverse: GCTGCAGCGTTATGTTGTT |
| *SmLAC35* | Forward: CCTACATGCTCCGCTTAATCA |
|  | Reverse: CTTCACGTAGGTGGCATCAA |
| *SmLAC36* | Forward: CGCCTTCCTCCTTCAACTATAC |
|  | Reverse: CCACTTCCACCGTCGTATTAT |
| *SmLAC37* | Forward: TCCAATCAAGACCGGAGAAAC |
|  | Reverse: AGCTTGTAGTTGACGACTTTGA |
| *SmLAC38* | Forward: CGTCGACCCCCGCTTCAGAACACC |
|  | Reverse: GACGGTGGAGGAAGCATCGTTTCG |
| *SmLAC39* | Forward: CGTTGATCCGCCGGTTCGGAATACC |
|  | Reverse: GTGGGAGATCAGCCGGCGGCGGC |
| *SmLAC40* | Forward: CCTGTTGAGAGGAACACCATAAG |
|  | Reverse: GGAAGATCTTTTGGAGGAGGCAAG |
| *SmLAC41* | Forward: GGCTATGACAGCAGCAAGAAGCATG |
|  | Reverse: CAGTGAATGGTTATATTTTGATCAG |
| *SmLAC42* | Forward: CGTTGGGGAACATGGTTTCCGTTCC |
|  | Reverse: CATGAGCCCAATAGCTTCGAAGAC |
| *SmLAC43* | Forward: CCGTCGGCGTTCCCACCGGAGGAT |
|  | Reverse: GGTGGGAGATCCTTAGGCGGCGGC |
| *SmLAC44* | Forward: CGGTTGCCGTTCCAAGAAGTGGATG |
|  | Reverse: GTTGAGGCTGCAGGGGGCGGTTCAC |
| *SmLAC45* | Forward: GTTGAGCATCCGATGCATTTACACG |
|  | Reverse: GATGATAACATGTTTTTTAACACCAG |
| *SmLAC46* | Forward: GGACCGTTATCAGATTTAAGGCTAAC |
|  | Reverse: CCTAAATACTAATGCTTCAGGGG |
| *SmLAC47* | Forward: CTTGATGCAGAACATAGCCGTGCGC |
|  | Reverse: CTACACGTAGCGTCGGAGTATCACAG |
| *SmLAC48* | Forward: CTGGTCGACCCGCCGTTGATGCAAAC |
|  | Reverse: GGCATATCCGGCGGAGGTGGCAAC |
| *SmLAC49* | Forward: GTTGCCGTTCCAAGAAATGGATGG |
|  | Reverse: GAGGCTGCAGGGGGCGCTTCACAG |
| *SmLAC50* | Forward: GAATTTCAACTTGGTGAACCCGCAG |
|  | Reverse: GCATTTGGGAAGATCTGCAGGTGG |
| *SmLAC51* | Forward: GGCGAGGGAAGCGATCATCCCATGC |
|  | Reverse: CTAAATAATAATTTCTGAGGGGGCGG |
| *SmLAC52* | Forward: CATCCCATGCATTTACATGGACACAG |
|  | Reverse: GAGGCTGCCGGAGGCGCTTCACAGC |
| *SmLAC53* | Forward: CATTGACCCGCCGTTGGGGAACATG |
|  | Reverse: GTTGAGGCTGTATGGGGCGGTTCAC |
| *SmLAC54* | Forward: CTGGTGGACCCACCTATGCGGAATA |
|  | Reverse: GGGGAAGATCTGGTGGAGGCGGCTG |
| *SmLAC55* | Forward: GGTGAATCCACAAGAGCGGAATACTG |
|  | Reverse: GCATTGTGGAAGATCCGCAGGAGG |
| *SmLAC56* | Forward: CCAAGAAATGGATGGAGCGCTATCAG |
|  | Reverse: CGAAATAATAACTGTTGAGGGGGCG |
| *SmLAC57* | Forward: GCCGTTCCATCGGGAGGATGGGTTG |
|  | Reverse: GGGCAAATCATTTGGAGGTGGCAG |
| *SmLAC58* | Forward: GGACCGCAATAAGATTTAAGGCAA |
|  | Reverse: GCAACGTGGCATATCCGGCGGAG |
| *SmLAC59* | Forward: CTCCGGGCCAAACCATAGATGTCTTG |
|  | Reverse: GTGAGCCTCTGTCGCTAAGCTTCGC |
| *SmLAC60* | Forward: CAGAAATTCAATCTCGTTGATCCTGT |
|  | Reverse: CATTTTGGCAAATCTTTAGGTGG |
| *SmLAC61* | Forward: GAGACGCGGCGGGTTTCAACCTCGTG |
|  | Reverse: CACGGCGGTAGATCAGGCGGCGGCG |
| *SmLAC62* | Forward: CGACCCAAACACGGATCCGCCAAAC |
|  | Reverse: GCGGTTCACAGAGGGGCATATCTGG |
| *SmLAC63* | Forward: CAAAACAGAGTTCCCAATGCCAG |
|  | Reverse: CCAAAGCCAGGCTCCCGCAGACAG |
| *SmLAC64* | Forward: GCACTGCCATTTCGAACGTCATTAC |
|  | Reverse: GTTCCAAACCGGAACCGTGTAAGTG |
| *SmLAC65* | Forward: GCACGGTGCAGGTGTACCCCAAG |
|  | Reverse: GGCCCTGCCGCAGAGGAGCGCGTTC |
| Sm-miR397.1 | Forward: TCATTGAGTGCAGCGTTGATGA |
| Sm-miR397.2 | Forward: TTGAGTGCAGCGTTGATGACA |
| Sm-miR408 | Forward: TGCACTGCCTCTTCCCTGGCT |
