# Supplementary material for: Analysis of the laccase gene family and miR397-/miR408-mediated posttranscriptional regulation in Salvia miltiorrhiza
Source: PeerJ. 2019 Aug 29;7:e7605. doi: 10.7717/peerj.7605 (PMC6717658; doi:10.7717/peerj.7605)
Supplement: Supplemental Information 8 [file peerj-07-7605-s008.docx]

**Table S5** Primers used for analysis of Sm-miR397-directed cleavage of *SmLACs*

| Gene name | Primer sequence (5’-3’) |
| --- | --- |
| *SmLAC3* | GSP: GGACCCTGACAGGTGCTACGGCTGGCAC |
|  | NGSP: GGCTTCTGAAGCTGGTTGTGAAGGC |
| *SmLAC23* | GSP: CGTTGTTCATGGACGCTGCTATCTTCATCC |
|  | NGSP: GCCGTGGTCATGTCTCTGGAGTTGGGC |
| *SmLAC50* | GSP: GATCCCGCTCGAGGCGTCGCAGAAGGAAAG |
|  | NGSP: GGTCATGTCTCTGGAGTTGGGCATTAC |
| *SmLAC55* | GSP: CCAATGCGGGCTACTGGTCAAACCGGTC |
|  | NGSP: GTGGTGGAGATGTTGACGTAGGGAGC |
| *SmLAC60* | GSP: GCACGTCGGGCACGGGTTGAGCCCGAG |
|  | NGSP: CGTCATGTTGTCCACCGCCACGATCATG |
