# Supplementary material for: Analysis of the laccase gene family and miR397-/miR408-mediated posttranscriptional regulation in Salvia miltiorrhiza
Source: PeerJ. 2019 Aug 29;7:e7605. doi: 10.7717/peerj.7605 (PMC6717658; doi:10.7717/peerj.7605)
Supplement: Supplemental Information 9 [file peerj-07-7605-s009.docx]

**Table S6** Primers used for analysis of Sm-miR408-directed cleavage of *SmLACs*

| Gene name | Primer sequence (5’-3’) |
| --- | --- |
| *SmLAC3* | GSP: CCCTCCTGGTCTAATAGGGCATTGAGTC |
|  | NGSP: CGCCGTTGTTCACTTCCAGCGTAGGC |
| *SmLAC18* | GSP: CATCAGCCCAAGGCGTTCGCATCTGCCG |
|  | NGSP: GAGGGTGTCGCCGTCTCTCGCCTCCAGAG |
| *SmLAC28* | GSP: CGATAAGTGTAACTTTTGCCAGCTCTAATTG |
|  | NGSP: GTGTCGCCGTTGTTCACCTCCAACGTTG |
