# Supplementary material for: Analysis of the laccase gene family and miR397-/miR408-mediated posttranscriptional regulation in Salvia miltiorrhiza
Source: PeerJ. 2019 Aug 29;7:e7605. doi: 10.7717/peerj.7605 (PMC6717658; doi:10.7717/peerj.7605)
Supplement: Supplemental Information 10 [file peerj-07-7605-s010.docx]

| Paralogous genes | | Ks | Ka | Ka/Ks |
| --- | --- | --- | --- | --- |
| *SmLAC63* | *SmLAC64* | 0.46 | 1.33 | 2.91 |
| *SmLAC63* | *SmLAC65* | 0.96 | 1.08 | 1.12 |
| *SmLAC64* | *SmLAC65* | 0.32 | 0.60 | 1.87 |
| *SmLAC45* | *SmLAC14* | 0.41 | 0.19 | 0.47 |
| *SmLAC45* | *SmLAC42* | 0.47 | 0.23 | 0.49 |
| *SmLAC14* | *SmLAC42* | 0.08 | 0.19 | 2.35 |
| *SmLAC22* | *SmLAC5* | 0.08 | 0.16 | 2.05 |
| *SmLAC56* | *SmLAC51* | 0.00 | 0.04 | n.a. |
| *SmLAC62* | *SmLAC53* | 0.00 | 0.00 | n.a. |
| *SmLAC20* | *SmLAC44* | 0.07 | 0.02 | 0.28 |
| *SmLAC44* | *SmLAC30* | 0.00 | 0.00 | n.a. |
| *SmLAC30* | *SmLAC20* | 0.07 | 0.02 | 0.28 |
| *SmLAC36* | *SmLAC35* | 0.31 | 0.35 | 1.16 |
| *SmLAC21* | *SmLAC46* | 0.06 | 0.04 | 0.64 |
| *SmLAC52* | *SmLAC33* | 0.81 | 0.22 | 0.27 |
| *SmLAC58* | *SmLAC48* | 0.19 | 0.22 | 1.21 |
| *SmLAC47* | *SmLAC41* | 0.14 | 0.24 | 1.75 |
| *SmLAC41* | *SmLAC27* | 0.00 | 0.00 | n.a. |
| *SmLAC27* | *SmLAC47* | 0.14 | 0.24 | 1.75 |
| *SmLAC37* | *SmLAC29* | 0.00 | 0.00 | n.a. |
| *SmLAC38* | *SmLAC9* | 0.00 | 0.00 | n.a. |
| *SmLAC25* | *SmLAC37* | 0.35 | 0.44 | 1.25 |
| *SmLAC25* | *SmLAC29* | 0.34 | 0.44 | 1.29 |
| *SmLAC25* | *SmLAC38* | 0.35 | 0.38 | 1.09 |
| *SmLAC25* | *SmLAC9* | 0.34 | 0.38 | 1.10 |
| *SmLAC39* | *SmLAC50* | 0.72 | 0.58 | 0.81 |
| *SmLAC28* | *SmLAC39* | 0.25 | 0.23 | 0.94 |
| *SmLAC28* | *SmLAC50* | 0.48 | 0.71 | 1.49 |
| *SmLAC18* | *SmLAC32* | 0.07 | 0.32 | 4.70 |
| *SmLAC32* | *SmLAC61* | 0.07 | 0.33 | 4.84 |
| *SmLAC61* | *SmLAC18* | 0.00 | 0.00 | n.a. |
| *SmLAC59* | *SmLAC55* | 0.00 | 0.50 | n.a. |
| *SmLAC55* | *SmLAC10* | 0.00 | 0.00 | n.a. |
| *SmLAC10* | *SmLAC59* | 0.57 | 0.55 | 0.96 |
| *SmLAC23* | *SmLAC24* | 0.57 | 0.57 | 0.99 |
| *SmLAC7* | *SmLAC13* | 0.66 | 0.39 | 0.59 |
| *SmLAC6* | *SmLAC15* | 0.23 | 0.19 | 0.84 |
| *SmLAC12* | *SmLAC31* | 0.53 | 0.48 | 0.91 |
| *SmLAC11* | *SmLAC57* | 0.00 | 0.00 | n.a. |
| *SmLAC4* | *SmLAC60* | 0.25 | 0.30 | 1.20 |
| *SmLAC60* | *SmLAC34* | 0.00 | 0.00 | n.a. |
| *SmLAC34* | *SmLAC4* | 0.24 | 0.29 | 1.21 |

**Table S7** Ka/Ks analysis for *SmLAC* paralogous genes from *S. miltiorrhiza*
