# Supplementary material for: Analysis of the laccase gene family and miR397-/miR408-mediated posttranscriptional regulation in Salvia miltiorrhiza
Source: PeerJ. 2019 Aug 29;7:e7605. doi: 10.7717/peerj.7605 (PMC6717658; doi:10.7717/peerj.7605)
Supplement: Supplemental Information 11 [file peerj-07-7605-s011.docx]

**Table S8** Numbers of *cis*-elements in promoter region of *SmLAC*s

| Element | ABRE | ERE | MRE | LTR | TC-rich | TGACG-motif | CGTCA-motif | TCA-element | MBS |
| --- | --- | --- | --- | --- | --- | --- | --- | --- | --- |
| Function | ABA response | Ethylene response | Light response | Low-temperature stress | Defense and stress | MeJA response | MeJA response | SA response | Drought stress |
| SmLAC1 | 3 | 1 | 1 |  |  | 3 | 3 | 1 |  |
| SmLAC2 | 3 |  | 1 | 2 |  | 3 | 2 | 1 |  |
| SmLAC3 | 9 | 3 | 2 |  |  | 2 | 2 | 1 |  |
| SmLAC4 | 8 | 2 | 1 | 1 |  | 1 | 2 | 2 |  |
| SmLAC5 | 2 |  | 1 | 1 |  | 2 | 2 | 2 |  |
| SmLAC6 | 2 | 1 |  |  |  |  | 2 | 1 |  |
| SmLAC7 | 2 |  |  | 1 |  |  |  | 1 |  |
| SmLAC8 | 5 | 2 |  | 2 |  |  | 1 | 1 | 1 |
| SmLAC9 | 6 | 2 |  | 2 |  |  |  | 1 | 1 |
| SmLAC10 | 4 |  | 1 | 2 |  | 1 | 1 | 1 | 1 |
| SmLAC11 | 7 |  |  | 1 |  | 1 | 1 |  | 1 |
| SmLAC12 | 1 | 1 |  |  |  | 1 | 1 | 1 | 1 |
| SmLAC13 | 3 | 2 |  |  | 1 |  |  |  | 1 |
| SmLAC14 | 2 |  |  |  |  |  | 1 | 1 |  |
| SmLAC15 | 2 | 1 |  |  |  |  |  |  |  |
| SmLAC16 | 6 |  | 1 | 1 |  |  |  | 1 |  |
| SmLAC17 | 1 |  | 1 |  |  |  |  |  |  |
| SmLAC18 |  | 1 |  | 3 |  |  |  |  |  |
| SmLAC19 | 6 | 2 | 1 | 1 |  | 1 | 1 | 1 |  |
| SmLAC20 | 3 |  |  |  |  |  | 1 | 1 |  |
| SmLAC21 | 6 | 5 |  |  | 1 |  | 2 | 1 | 1 |
| SmLAC22 | 4 | 1 |  | 1 |  | 1 | 1 | 1 | 1 |
| SmLAC23 | 3 | 3 |  |  |  | 1 | 1 | 1 | 1 |
| SmLAC24 |  | 4 |  |  |  | 1 | 1 | 1 | 1 |
| SmLAC25 |  |  |  | 1 |  | 1 | 1 | 1 | 1 |
| SmLAC26 | 3 |  |  |  |  | 3 | 3 | 1 | 1 |
| SmLAC27 | 3 |  | 1 |  |  | 3 | 3 | 1 | 1 |
| SmLAC28 | 3 | 3 | 1 |  |  | 2 | 2 | 1 | 1 |
| SmLAC29 | 7 | 2 | 1 |  |  | 1 |  | 1 | 1 |
| SmLAC30 | 1 |  |  | 1 |  | 2 | 2 | 1 | 1 |
| SmLAC31 | 2 | 1 |  |  |  | 1 | 1 | 1 |  |
| SmLAC32 | 2 |  |  | 1 |  |  |  | 1 |  |
| SmLAC33 |  |  |  |  |  |  | 1 | 1 |  |
| SmLAC34 |  | 2 |  | 1 |  | 1 | 1 |  |  |
| SmLAC35 | 3 | 3 | 1 |  |  | 2 | 2 | 1 | 1 |
| SmLAC36 |  | 2 |  |  |  |  |  | 1 | 1 |
| SmLAC37 | 6 | 2 |  | 2 |  | 1 |  | 1 |  |
| SmLAC38 | 6 | 1 |  |  |  |  |  | 1 | 1 |
| SmLAC39 | 4 |  |  |  | 1 | 3 | 3 | 1 |  |
| SmLAC40 | 5 |  | 1 |  |  | 1 | 1 | 1 |  |
| SmLAC41 | 1 | 1 |  |  |  | 1 | 1 | 1 | 1 |
| SmLAC42 | 1 | 1 |  |  |  | 1 | 1 | 1 | 1 |
| SmLAC43 | 2 | 3 |  |  | 1 | 1 | 1 | 1 |  |
| SmLAC44 | 3 |  |  |  |  | 3 | 3 | 1 |  |
| SmLAC45 | 1 | 2 |  | 1 |  | 2 | 2 | 1 | 1 |
| SmLAC46 | 3 | 1 | 1 |  | 1 | 1 | 1 | 1 |  |
| SmLAC47 | 3 | 1 |  |  |  | 1 | 1 | 1 |  |
| SmLAC48 | 7 | 1 |  |  |  | 2 | 2 | 1 | 1 |
| SmLAC49 | 4 | 1 |  | 1 |  | 1 | 1 | 1 |  |
| SmLAC50 | 4 | 1 |  | 1 |  | 1 | 1 | 1 |  |
| SmLAC51 | 3 |  |  |  | 1 | 3 | 3 | 1 |  |
| SmLAC52 |  | 1 | 1 |  |  |  |  | 1 | 1 |
| SmLAC53 | 9 | 5 |  |  | 1 |  |  |  |  |
| SmLAC54 | 3 |  | 1 |  | 1 | 2 | 2 |  |  |
| SmLAC55 | 3 |  |  | 2 |  | 1 | 1 |  | 1 |
| SmLAC56 | 2 | 2 |  | 2 |  | 2 | 2 |  |  |
| SmLAC57 | 6 | 2 |  | 1 |  | 1 | 1 | 1 |  |
| SmLAC58 | 5 | 2 |  | 1 |  | 1 | 1 | 1 | 1 |
| SmLAC59 | 3 | 2 |  | 1 |  | 1 | 1 | 1 | 1 |
| SmLAC60 |  | 4 |  |  |  | 1 | 1 | 1 | 1 |
| SmLAC61 |  |  |  |  |  | 1 | 1 | 1 | 1 |
| SmLAC62 | 3 |  |  |  |  | 1 | 1 | 1 | 1 |
| SmLAC63 | 2 |  |  | 1 | 1 | 2 | 2 | 1 | 1 |
| SmLAC64 | 7 | 3 |  | 1 | 3 |  |  | 1 | 1 |
| SmLAC65 | 1 | 2 | 1 |  |  | 2 | 2 | 1 | 1 |
