# Supplementary material for: Analysis of the laccase gene family and miR397-/miR408-mediated posttranscriptional regulation in Salvia miltiorrhiza
Source: PeerJ. 2019 Aug 29;7:e7605. doi: 10.7717/peerj.7605 (PMC6717658; doi:10.7717/peerj.7605)
Supplement: Supplemental Information 12 [file peerj-07-7605-s012.docx]

**Table S9** PCR efficiency of each *SmLAC* qRT-PCR primer

| **Gene name** | **Amplification efficiency（%）** | **Regression coefficient** | **Standard Curve** |
| --- | --- | --- | --- |
| *SmLAC26* | 101.6 | 0.9993 |  |
| *SmLAC28* | 93.6 | 0.9957 |  |
| *SmLAC34* | 92.7 | 0.9998 |  |
| *SmLAC41* | 110 | 0.9968 |  |
| *SmLAC46* | 105.8 | 0.9977 |  |
