# Supplementary material for: Analysis of the laccase gene family and miR397-/miR408-mediated posttranscriptional regulation in Salvia miltiorrhiza
Source: PeerJ. 2019 Aug 29;7:e7605. doi: 10.7717/peerj.7605 (PMC6717658; doi:10.7717/peerj.7605)
Supplement: Supplemental Information 13 [file peerj-07-7605-s013.docx]

| **Gene** | **Go Term ID** | **Go Term** | **Ontology** | **P-value** |
| --- | --- | --- | --- | --- |
| SmLAC63, 64, 65 | GO:0005618 | cell wall | Cellular component | 0.000382323 |
| SmLAC63 | GO:0005774 | vacuolar membrane | Cellular component | 0.001341105 |
| SmLAC63 | GO:0005886 | plasma membrane | Cellular component | 0.001809244 |
| SmLAC63, 64, 65 | GO:0009505 | plant-type cell wall | Cellular component | 0.006001615 |
| SmLAC63, 65 | GO:0009506 | plasmodesma | Cellular component | 0.000221245 |
| SmLAC6, 7, 13, 15, 16 | **GO:0009698** | phenylpropanoid metabolic process | Biological process | 0.000382451 |
| SmLAC4, 6, 7, 8,11, 12, 13, 15, 16, 25, 29, 31, 34, 37, 38, 43, 57, 59, 60 | **GO:0009809** | lignin biosynthetic process | Biological process | 0.005624675 |
| SmLAC4, 11, 12, 31, 34, 40, 57, 60 | **GO:0009834** | plant-type secondary cell wall biogenesis | Biological process | 0.000424180 |
| SmLAC63 | GO:0009932 | cell tip growth | Biological process | 0.000753293 |
| SmLAC8, 25, 29, 37, 38, 59 | **GO:0010023** | proanthocyanidin biosynthetic process | Biological process | 0.000484391 |
| SmLAC1, 10, 23, 24, 50, 55 | GO:0010228 | vegetative to reproductive phase transition of meristem | Biological process | 0.002176483 |
| SmLAC63, 65 | GO:0016020 | membrane | Cellular component | 0.000191616 |
| SmLAC4, 6, 7, 11, 12, 13, 15, 16, 31, 34, 40, 57, 60 | GO:0016491 | oxidoreductase activity | Molecular function | 0.000123606 |
| SmLAC63 | GO:0046658 | anchored component of plasma membrane | Cellular component | 0.000728944 |
| SmLAC1, 3, 8, 10, 18, 23, 24, 25, 26, 28, 29, 32, 37, 38, 39, 50, 54, 55, 59 | GO:0046688 | response to copper ion | Molecular function | 0.000326217 |
| SmLAC63, 65 | GO:0048046 | apoplast | Cellular component | 0.000126183 |
| SmLAC64 | GO:0080167 | response to karrikin | Biological process | 0.005624632 |

**Table S10** Gene Ontology terms of SmLACs
