# Supplementary material for: Analysis of the laccase gene family and miR397-/miR408-mediated posttranscriptional regulation in Salvia miltiorrhiza
Source: PeerJ. 2019 Aug 29;7:e7605. doi: 10.7717/peerj.7605 (PMC6717658; doi:10.7717/peerj.7605)
Supplement: Supplemental Information 14 [file peerj-07-7605-s014.docx]

**Table S10** Summary in secondary metabolism of LAC

| **Gene name** | **Organism** | **Expression pattern** | **Putative function** | **References** |
| --- | --- | --- | --- | --- |
| *AtLAC4* | *A. thaliana* | high expression in stems | lignin biosynthesis | Berthet et al., 2011; Zhao et al., 2013 |
| *AtLAC10* | *A. thaliana* | high expression in stems | lignin biosynthesis |  |
| *AtLAC11* | *A. thaliana* | high expression in stems | lignin biosynthesis | Zhao et al., 2013 |
| *AtLAC15* | *A. thaliana* | high expression in seeds | flavonoids | Cai et al., 2006 |
| *AtLAC17* | *A. thaliana* | high expression in stems | lignin biosynthesis | Berthet et al., 2011; Zhao et al., 2013 |
| *PtLAC1* | *P. trichocarpa* | high expression in stems | lignin biosynthesis | Lu et al., 2013 |
| *PtLAC2* | *P. trichocarpa* | high expression in xylems | lignin biosynthesis | Lu et al., 2013 |
| *PtLAC14* | *P. trichocarpa* | high expression in xylems | lignin biosynthesis | Lu et al., 2013 |
| *PtLAC15* | *P. trichocarpa* | high expression in xylems | lignin biosynthesis | Lu et al., 2013 |
| *PtLAC20* | *P. trichocarpa* | high expression in xylems | lignin biosynthesis | Lu et al., 2013 |
| *PtLAC23* | *P. trichocarpa* | high expression in xylems | lignin biosynthesis | Lu et al., 2013 |
| *PtLAC24* | *P. trichocarpa* | high expression in xylems | lignin biosynthesis | Lu et al., 2013 |
| *PtLAC40* | *P. trichocarpa* | high expression in xylems | lignin biosynthesis | Lu et al., 2013 |
| *PtLAC41* | *P. trichocarpa* | high expression in stems | lignin biosynthesis | Lu et al., 2013 |
| *PtLAC49* | *P. trichocarpa* | high expression in xylems | lignin biosynthesis | Lu et al., 2013 |
| *PbLAC1* | *P. bretschneideri* | high expression in stems and fruits | lignin biosynthesis | Chen et al., 2019 |
| *PbLAC6* | *P. bretschneideri* | high expression in fruits | lignin biosynthesis | Chen et al., 2019 |
| *PbLAC29* | *P. bretschneideri* | high expression in fruits | lignin biosynthesis | Chen et al., 2019 |
| *PbLAC36* | *P. bretschneideri* | high expression in fruits | lignin biosynthesis | Chen et al., 2019 |
| BdLAC5 | *B. distachyon* | high expression in  stems | lignin biosynthesis | Wang et al., 2015 |
| SofLAC | *Saccharum* spp | high expression in stems | lignin biosynthesis | Cesarino et al., 2013 |
|  |  |  |  |  |
